# Effector FpECIR from *Fusarium pseudograminearum* targets wheat ethylene signaling pathway to suppress plant immunity

**DOI:** 10.1007/s44154-026-00311-7

**Published:** 2026-07-02

**Authors:** Jiangang Kang, Yun Li, Limin Wang, Zhifang Wang, Cong Chen, Xinlong Wang, Ziming Wang, Shengli Ding, Honglian Li, Haiyang Li

**Affiliations:** 1 College of Plant Protection, Henan Agricultural University, Zhengzhou, 450002 China; 2 Postdoctoral Station of Crop Science, Henan Agricultural University, Zhengzhou, 450002 China

**Keywords:** *Fusarium pseudograminearum*, FpECIR, TaPLATZ2B, Transcriptome analysis

## Abstract

*Fusarium pseudograminearum* (*F. pseudograminearum*) is the main causal agent of Fusarium Crown Rot (FCR) posing a severe threat to wheat yield. Effectors, as one class of weapons of phytopathogens, help pathogens infect their hosts by disturbing plant immunity. In this study, we identified a secreted effector, FpECIR, which has no functional domains, suppresses INF1-triggered cell death in *Nicotiana benthamiana*. Once translocated into wheat cells, FpECIR (without signal peptide) interacts with TaPLATZ2B, and silencing *TaPLATZ2B* and its alleles makes wheat more susceptible to *F. pseudograminearum*. Deletion of residues 29-35 of FpECIR disrupted its interaction with TaPLATZ2B, and eliminated its ability to suppress INF1-induced cell death. The pathogenicity of deletion mutants Δ*Fpecir* on wheat coleoptiles was significantly reduced. Analysis of transcriptome data showed that infection with the Δ*Fpecir* strain triggered the upregulation of defense-related genes in wheat coleoptiles, many of which were functionally enriched in hormone signaling pathways. qRT-PCR results revealed that *FpECIR* suppressed the expression of *TaPLATZ2B* and of genes related to ethylene signaling pathway. Furthermore, EMSA experiment result proved that FpECIR can inhibit the ability of TaPLATZ2B binding with the promoter region of ethylene response factors (*TaERF020L*). Suppressing the transcription levels of genes related to ethylene biosynthesis made the wheats more susceptible to FCR. Overall, the results showed that *FpECIR* plays an important role in the pathogenicity of *F. pseudograminearum*, and that the integrity of FpECIR is necessary for its interaction with TaPLATZ2B and for its suppression of INF1-induced cell death. Additionally, FpECIR not only affects the expression of *TaPLATZ2B* but also inhibits the function of TaPLATZ2B to regulate plant defense response through ethylene signaling pathways.

**Supplementary Information:**

The online version contains supplementary material available at 10.1007/s44154-026-00311-7.

## Introduction

Wheat is one of the major staple grains worldwide, serving as a primary source of dietary energy and protein for a large proportion of the global population and providing approximately 40% of total caloric and protein intake. China is a major wheat production country, accounting for approximately 17% of global wheat production (Li et al. 2019; Shi et al. 2024). However, Fusarium Crown Rot (FCR), which is primarily caused by the soil-borned pathogenic fungus *Fusarium pseudograminearum*, was first identified in Henan province of China in 2011, and can lead to substantial yield reductions around the world (Deng et al. 2019; Li et al. 2012; Smiley et al. 2007). Additionally, deoxynivalenol (DON) and other mycotoxins are produced by *F. pseudograminearum* during the pathogen infection process. These mycotoxins can contaminate grains and derived products, thereby compromise grain quality and pose serious risks to human and animal health (Obanor and Chakraborty 2014; Xu et al. 2024). Given the global importance of wheat production, surveys about the distribution of FCR in China from 2013 to 2016 were conducted and revealed that the disease was widespread throughout the HuangHuai wheat-growing region, which encompasses seven of Chinese most important wheat-producing provinces (Zhou et al. 2019). Recently, several FCR resistance genes have been identified. For instance, TaCAT2, a catalase antioxidant enzyme, can enhance wheat resistance to FCR by scavenging reactive oxygen species (ROS). Furthermore, TaSnRK1α interacts with and phosphorylates TaCAT2 at specific sites, thereby enhancing its ability to eliminate ROS and conferring wheat resistance to FCR (Yang et al. 2025b). Overexpression *TaCWI*, a gene encoding a cell wall invertase, significantly increases cell wall thickness and associated components to improve wheat resistance against FCR (Lv et al. 2023). Although several quantitative trait loci (QTLs) associated with FCR resistance have been identified in wheat, only a limited number are effective (Pariyar et al. 2020).

During the infection process, interactions between pathogens and hosts facilitate pathogen colonization of host tissues. In the case of *F. pseudograminearum*, various products are generated during the infection stage, including secondary metabolites, toxins, effectors and enzymes (Kazan and Gardiner 2018). The canonical toxin DON acts as a virulence factor participating in the infection process of *F. pseudograminearum*. Inoculation of wheat leaves with DON can trigger the accumulation of hydrogen peroxide and even induce cell death, while the expression levels of several defense genes are simultaneously up-regulated (Cheng et al. 2015). *TRI5* is one of key genes involved in DON biosynthesis. The pathogenicity of the knock out mutants Δ*TRI5* in different *F. pseudograminearum* strains was significantly reduced compared to that of wild type strains (Knight and Sutherland 2016). Plants produce a number of phytoalexins in response to phytopathogens infection. However, Fusarium Detoxification of Bx2 (FDB2) produced by *F. pseudograminearum* can degrade benzoxazolinone into non-toxic compound, thereby facilitating pathogen infection (Kettle et al. 2015; Zhao et al. 2024). Recently, researchers found that the effector Fp00392 not only promotes *F. pseudograminearum* infection, but also function as a pathogen-associated molecular pattern (PAMP) to trigger plant immunity (Yang et al. 2025a). Secretome analysis of *F. pseudograminearum* revealed that an apoplastic effector named FpCDP1 could induce cell death and other immune responses. The function of FpCDP1 is conserved in other species, and its homologs exhibit similar functions. Therefore, FpCDP1 may also function as a PAMP across different fungal species (Liu et al. 2025). Nevertheless, the targets of these effectors remain unidentified and elucidating them is crucial for understanding the pathogenic mechanisms of *F. pseudograminearum*.

Transcription factors (TFs) are key regulators that participate in diverse plant signal transduction pathways by activating or repressing the expression of downstream target genes (Franco-Zorrilla et al. 2014; Jin et al. 2017). Plant AT-rich sequence- and zinc-binding (PLATZ) proteins, representing a novel class of TFs, were first identified in peas and could bind A/T-rich DNA sequences (Nagano et al. 2001). PLATZ proteins have been shown to participate broadly in plant growth, development, and stress responses. In various plant species, PLATZ genes exhibit diverse functions. In barley, 11 *HvPLATZ* genes have been identified, displaying differential expression patterns in response to drought or salt stress (Feng et al. 2024). In tomato, 24 *SlPLATZs* genes are classified into four distinct groups, and are involved in tissue development as well as responses to salt stress (Zhang et al. 2023b). In Arabidopsis, the *ORE15* genes encods a PLATZ TF that plays a key role in leaf growth and senescence, and deletion of *ORE15* results in enlarged leaf size and an extended lifespan(Kim et al. 2018). Similarly, *ClPLATZ* genes from watermelon are involved in regulating tolerance to abiotic stress, as well as plant growth and development. Moreover, some of these genes contribute to their hosts resistance against fungal infections (Qi et al. 2023). In wheat, which has a complex genome, 62 *TaPLATZ* genes were characterized and classified into six phylogenetic groups (Fu et al. 2020). In response to saline-alkali stress, TaWRKY55 directly upregulates the expression of *TaPLATZ2*, which suppresses the expression of *TaHA2/TaSOS3*, and enhances sensitivity of wheat to the abiotic stress (Wei et al. 2024). In the elongating stems and developing spikes of wheat, the expression of *TaPLATZ-A1*(*RHT25*) showed an upward trend. The function of RHT25 is associated with wheat height by modulating the effect of DELLA (Zhang et al. 2023a). Except for the similar functions of *PLATZ* genes in other species, the histone acetyltransferase TaHAG1 confers wheat resistance to powdery mildew by upregulating the expression of *TaPAD4*. This process requires the participation of TaPLATZ5, in which TaHAG1 interacts with TaPLATZ5 to activate *TaPAD4* expression (Song et al. 2022). Collectively, these findings demonstrate that PLATZ transcription factors are critical regulators of plant growth and development and play essential roles in response to both abiotic and biotic stresses. The conserved and species-specific functions of PLATZ proteins underscore their potential as targets for crop improvement in wheat and other important crops.

In this study, we identified an effector FpECIR (*F. pseudograminearum* Effector Containing two Internal Repeats) from *F. pseudograminearum* whose encoding gene is upregulated during the infection stage. Knockout mutants Δ*Fpecir* exhibited significantly reduced pathogenicity of *F. pseudograminearum* on wheat. FpECIR localized in both nucleus and cytoplasm and was able to suppress INF1-induced cell death. Furthermore, FpECIR interacts with the wheat transcription factor TaPLATZ2B, thereby modulating the expression of ethylene signaling pathway related genes. Barley stripe mosaic virus (BSMV) mediated silencing of *TaPLATZ2B* enhanced wheat susceptibility to *F. pseudograminearum* infection, and it may also affect normal wheat development.

## Results

### FpECIR functions as a cytoplasm effector

**Fig. 1:**
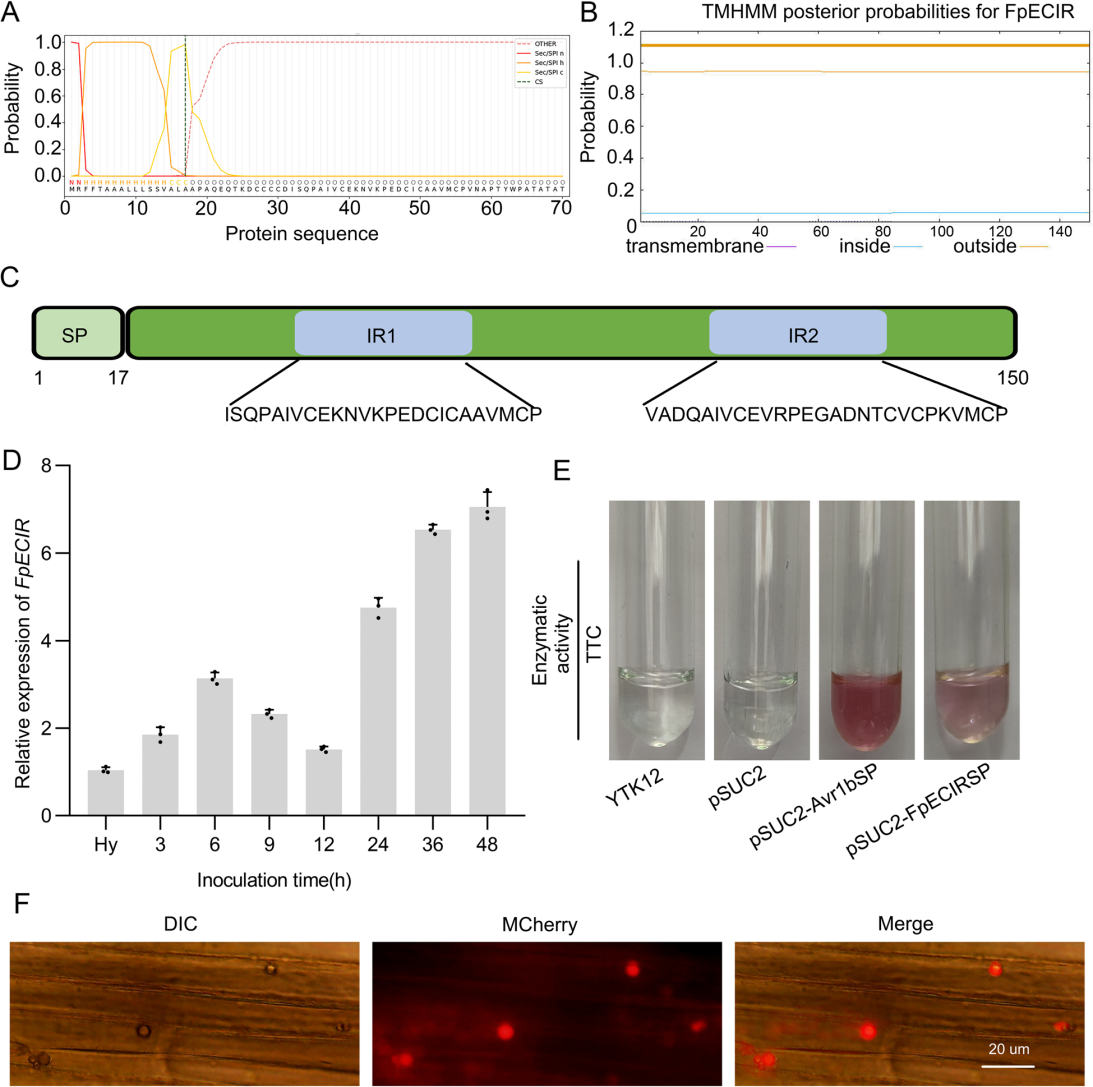
FpECIR functions as a cytoplasmic effector from *F. pseudograminearum*. **A** Signal peptide of FpECIR with 17 amino acids residues was predicted using SpgnalP 6.0. **B** No transmembrane domain of FpECIR was predicted using TMHMM-2.0. **C** Schematic drawing of FpECIR showing the distribution of signal peptide and two internal repeats (IR1 and IR2) predicted by SMART. **D** The expression pattern of *FpECIR* during *F. pseudograminearum* infected wheat seedlings was validated by qRT-PCR, and the data were calculated using the 2^-ΔΔCt^ method. Hyphae (Hy) was used as a control. Infected wheat coleoptiles samples were harvested at different infected times (3, 6, 9, 12, 24, 36 and 48 h) for RNA extraction. *FpECIR* showed upregulated trend during infection stage. Experiments were repeat three times. **E** YTK12 yeast system was used for functional validation of the signal peptide by detecting the invertase enzymatic activity. Yeast YTK12 cultures carrying pSUC2-*FpECIRSP* or pSUC2-*Avr1bSP* (positive control) can make the colorless 2, 3, 5-triphenyltetrazolium chloride (TTC) to the insoluble red 1, 3, 5-triphenylformazan (TPF), whereas cultures carrying the negative controls YTK12 and pSUC2 are not. **F** Wheat seedlings were infected with the transformants expressing *FpECIR*: *NLS *fusion genes and the red fluorescence signal were observed in the nuclei. Scale bar, 20 μm

FpECIR (*F. pseudograminearum* Effector Containing two Internal Repeats) is encoded by *F. pseudograminearum 08419* from the *F. pseudograminearum* strain WZ-8A. The amino acid sequences show high similarity to FPSE_01532 from the strain *F. pseudograminearum* CS3096 (Figure S1A). Meanwhile, NCBI BLAST analysis identified a homologous protein FGSG_06712, in the strain *F. graminearum* PH-1, has been reported as a candidate cytoplasm effector (Hao et al. 2020), but its function was not characterized so far. The amino acid sequence of FpECIR showed 95% identity with the sequence encoded by *FGSG_06712* (Figure S1B), suggesting that FpECIR is also likely to function as a cytoplasmic effector. FpECIR consists of 150 amino acids, which is encoded by a 453 bp open reading frame (ORF). To characterize its structural features, the amino acid sequence of FpECIR was analyzed using multiple bioinformatic tools. The sequence was predicted to contain a 17-amino-acid signal peptide, but no transmembrane helices were predicted (Fig. 1A and B). Furthermore, no known conserved functional domains were detected in FpECIR; however, two internal repeat regions, designated IR1 and IR2, were identified within the protein sequence (Fig. 1C).

To further investigate the function of *FpECIR*, its expression pattern during the infection process was examined. Samples were collected from different infection points (3, 6, 9, 12, 24, 36 and 48 h post inoculation), as well as from fungal hyphae, and transcript levels were quantified by qRT-PCR. The results showed that *FpECIR* expression was markedly upregulated during infection, with transcript levels increasing by more than ten folds compared with those in hyphae, indicating that *FpECIR* may play a crucial role during *F. pseudograminearum* infection process (Fig. 1D).

To test the function of the FpECIR signal peptide, the YTK12 yeast secretion system was used to examine the enzymatic activity of the secreted invertase encoded by the pSUC2 plasmid. In this assay, the recombinant plasmid pSUC2-*FpECIRSP* was generated and transformed into YTK12 yeast strain. Invertase activity was detected by observing the color change of TTC solution. Results showed that yeast YTK12 cultures transformed with pSUC2 or only YTK12 culture (negative control) failed to induce TTC color conversion. In contrast, yeast YTK12 cultures carrying pSUC2-*FpECIRSP* or pSUC2-*Avr1bSP* (positive control) converted the colorless TTC solution to red (Fig. 1E). These results suggests that the signal peptide of FpECIR functions as a secretory signal.

Fungal effectors that interfere with host immunity can be classified as cytoplasmic or apoplastic effectors, depending on whether they are translocated into or outside plant cells, respectively. To investigate whether FpECIR can be translocated into plant cells, the reconstructed vector P_FpECIR_-*FpECIR*-mCherry-3 ×*NLS* was generated and transformed into the wild type strain WZ-8A. In wheat coleoptiles infected with transformants expressing the fusion gene, red fluorescence signals were observed in the nucleus of wheat cells at the inoculation sites (Fig. 1F). These foundings provide the evidence that FpECIR can be translocated into wheat cells and functions as a cytoplasmic effector.

### FpECIR can suppress the INF1 triggered cell death in *N. benthamiana*

**Fig. 2:**
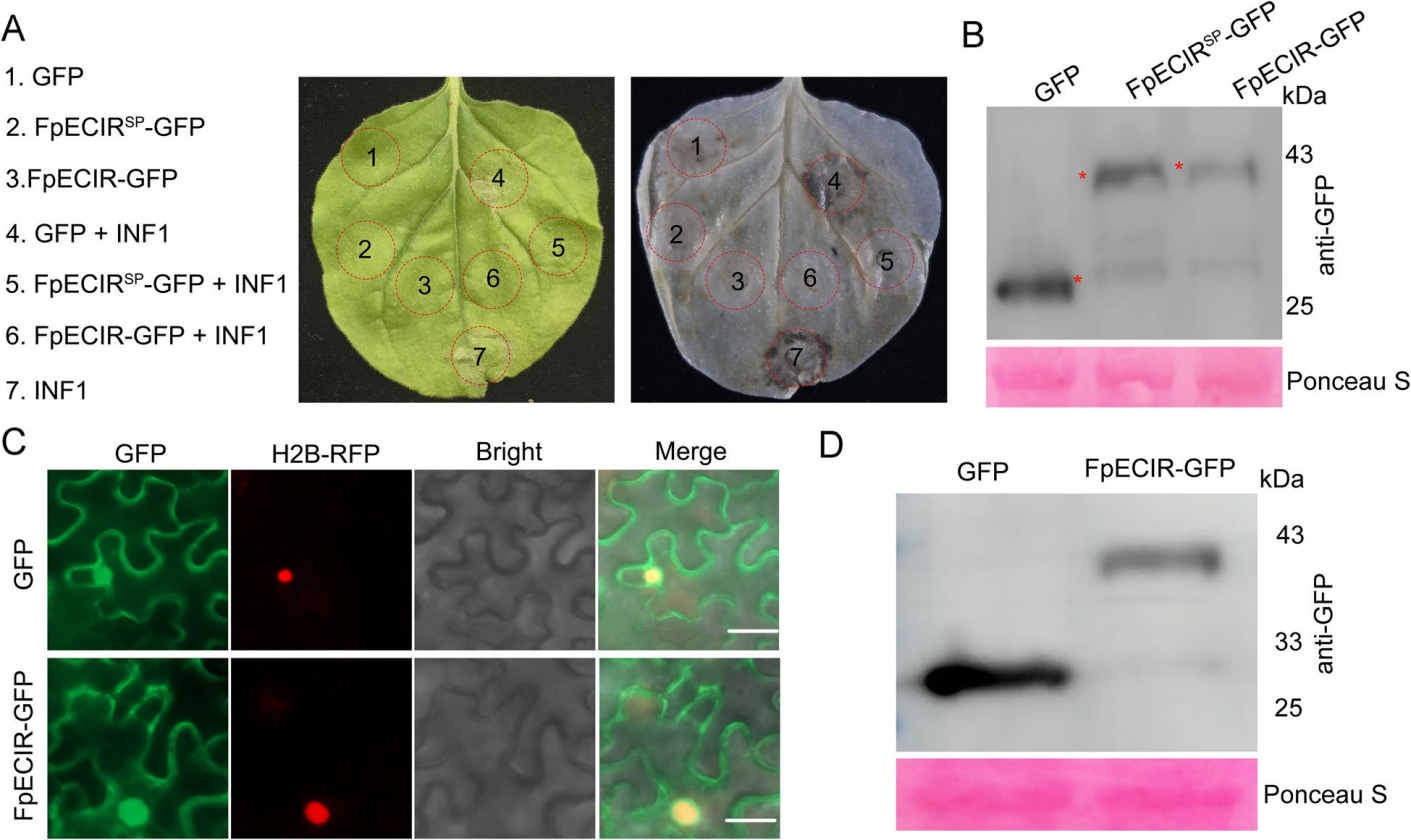
FpECIR localized in cell membrane and nucleus to suppress INF1-triggered cell death. **A** Transient expression *FpECIR*^SP^ and *FpECIR* in *N.* *benthamiana* leaves 24 h prior to infiltration of the INF1-carrying strain GV3101. Like the negative control, expressing *GFP* alone or fusion genes *FpECIR*^SP^-*GFP* and *FpECIR*-*GFP* can not induce cell death, while unlike *GFP*, expressing *FpECIR*^SP^ and *FpECIR* both can suppress INF1 induced cell death which function as positive control. Middle picture presents schematic drawing of the infiltration sites. The right picture represents the infiltrated leaf decolored by ethanol. **B** Western blot and immunoblot analysis of the protein expressing GFP, FpECIR^SP^-GFP and FpECIR-GFP. **C** Transient expression GFP or FpECIR-GFP in the transgenic *N.* *benthamiana* leaves expressing H2B-RFP, the fluorescence signals were observed 2 days post inoculation. Like the subcellular localization of GFP, FpECIR-GFP also localized in cell nucleus and cytoplasm. **D** Western blot and immunoblot analysis of the protein expressing GFP and FpECIR-GFP in *N.* *benthamiana* leaves

To investigate the role of FpECIR in modulating plant immunity, *FpECIR*^*SP*^ (full length) and *FpECIR* (without signal peptide) were transiently expressed in *N. benthamiana*. Previous results showed that FpECIR can be secreted and translocated into plant cells. To further assess its immunomodulatory activity and the requirement of its signal peptide, two fusion gene constructs PVX-*FpECIR*^SP^-*GFP* and PVX-*FpECIR*-*GFP* were generated. *Agrobacterium*-mediated transient expression assays were performed by infiltrating *N. benthamiana* leaves with *Agrobacterium* strains carrying each construct, either alone or 24 h prior to infiltration with an INF1-carrying strain. At 6 days post-infiltration, the positive control INF1 successfully induced cell death. As with the negative control GFP, neither FpECIR^SP^ nor FpECIR induced cell death; however, both fusion genes were able to suppress INF1 triggered cell death (Fig. 2A). Expression of these genes were confirmed by western blot (Fig. 2B). These results suggested that the signal peptide was not necessary for the function of FpECIR, and that the effector was able to suppress plant immunity.

Given that the signal peptide of FpECIR was not necessary for its function in *N. benthamiana*, the subcellular localization of FpECIR was further investigated. *GFP* alone and *FpECIR*-*GFP* fusion gene were expressed via *A. tumefaciens* mediated transient expression in the transgenic *N. benthamiana* leaves expressing H2B-RFP, which function as the nucleus marker. At 2 days post-inoculation (dpi), the green fluorescence signal of FpECIR was observed in nucleus and cytoplasm using confocal microscopy, exhibiting a distribution pattern similar to that of GFP alone (Fig. 2C). Western blot was used to examine the GFP and GFP-FpECIR proteins (Fig. 2D). At the same time, the FpECIR with nucleus export signals (NES) reconstructed vector-PVX-*FpECIR*-*NES*-*GFP* was generated and transiently expressed in *N.* *benthamiana* leaves to test if this mutant can function as *FpECIR*. 24 h prior to infiltration with an INF1-carrying strain, transient expressing GFP or FpECIR-NES-GFP cannot suppress INF1 triggered cell death, while expressing *FpECIR* still can inhibit INF1 triggered cell death, Western blot was used to examine the GFP and GFP-FpECIR proteins (Figure S2). These results indicate the nucleus localization is important for the function of FpECIR.

### The integrity of the sequence was essential for the function of FpECIR

**Fig. 3:**
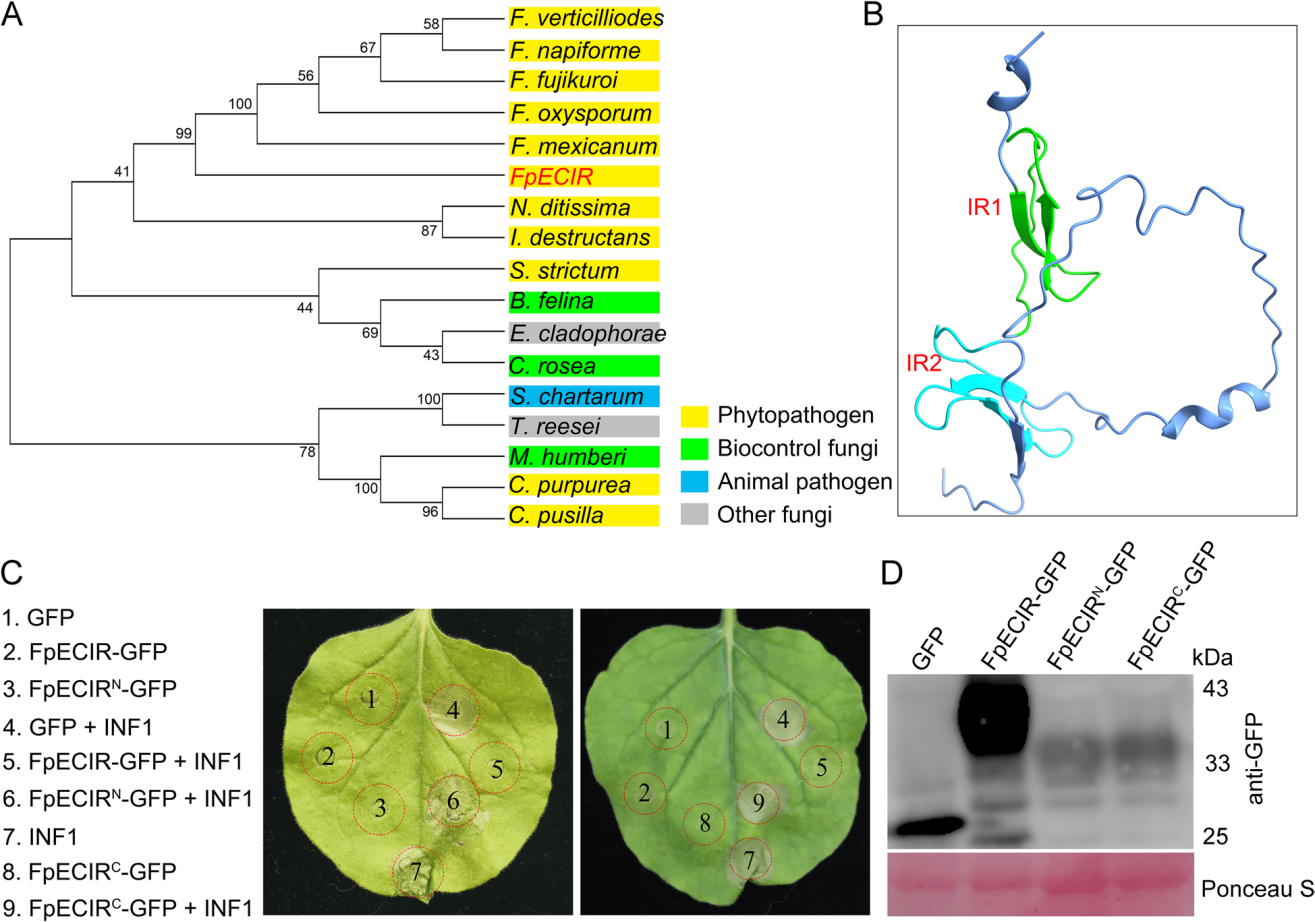
Sequence integrity is required for the function of FpECIR. **A** The phylogeny tree was used to systemically analyze the homologs of FpECIR and its conservation in selected species. Homologs distributed in phytopathogens, animal pathogens, biocontrol fungi and other fungi. **B** Structural model of FpECIR, the predicted structure was visualized with ChimeraX and N-terminal region C-terminal region were structurally similar. **C**-**E** Transient expression PVX-*FpECIR*^*N*^-*GFP* and PVX-*FpECIR*^*C*^-*GFP* in *N.* *benthamiana* leaves 24 h prior to infiltration of the INF1-carrying strain GV3101, both of them lost the function to suppress INF1-triggered cell death like the full-length FpECIR. **F** Western blot and immunoblot analysis of the protein expressing GFP and FpECIR-GFP, FpECIR^N^-GFP and FpECIR^C^-GFP in *N.* *benthamiana* leaves

To systemically analyze the conservation of FpECIR among fungi, different species of representative fungi belonging to Ascomycota and Basidiomycota were selected. Notably, homologs of FpECIR were absent in several well-characterized phytopathogens, including *Magnaporthe oryzae*, *Sclerotinia sclerotiorum*, *Botrytis cinerea*, *Ustilago maydis* and *Puccinia striiformis*. The species containing homologs genes are mainly plant pathogens, but also include animal pathogens, biocontrol fungi, and other fungi (Fig. 3A).

Previous analysis revealed that the amino acid sequence of FpECIR contains two internal repeats. Multiple sequence alignment of the amino acid sequences encoded by these homologs showed that significant divergence across the full-length sequences, though most sequences had the two internal repeats (Fig. 3B and S3). The predicted protein structure of FpECIR by AlphaFold v3 showed that N terminal regions (18–67 aa) and C terminal regions (68–150 aa) had the similar three-dimensional structures (Fig. 3B). Both regions contained an internal repeat, promoting further investigation into which region is responsible for the function of FpECIR. Reconstructed PVX-*FpECIR*^*N*^-*GFP* and PVX-*FpECIR*^*C*^-*GFP* were transiently expressed in *N. benthamiana* leaves to test their ability to suppress INF1 induced cell death. The results showed that neither *FpECIR*^*N*^ nor *FpECIR*^*C*^ was able to suppress INF1 induced cell death (Fig. 3C). The expression of *FpECIR*-*GFP*, *FpECIR*^*N*^-*GFP* and *FpECIR*^*C*^-*GFP* in *N.* *benthamiana* leaves was confirmed by Western blot (Fig. 3D). These results indicat that the N-terminal internal repeat is conserved among the homologs of FpECIR, that the integrity of the full FpECIR’ sequence is required for its function.

### FpECIR interacts with the transcription factor TaPLATZ2B

To screen for potential FpECIR-interacting proteins, a cDNA library was constructed from RNA extracted from *F. pseudograminearum*-infected wheat coleoptiles. Yeast two hybrid (Y2H) assays were performed using FpECIR as bait in five independent screens, and fourteen candidates interacting proteins were identified (Table S2). Sequencing analysis indicated that three of the clones were a putative wheat AT-rich zinc-binding protein. This protein is encoded by *TraesCS2B02G469000*, an allele of which was reported to encode a protein called TaPLATZ5, that plays an important role in protecting wheat against powdery mildew (Song et al. 2022). Consequently, the protein was designated TaPLATZ2B in this study.

**Fig. 4:**
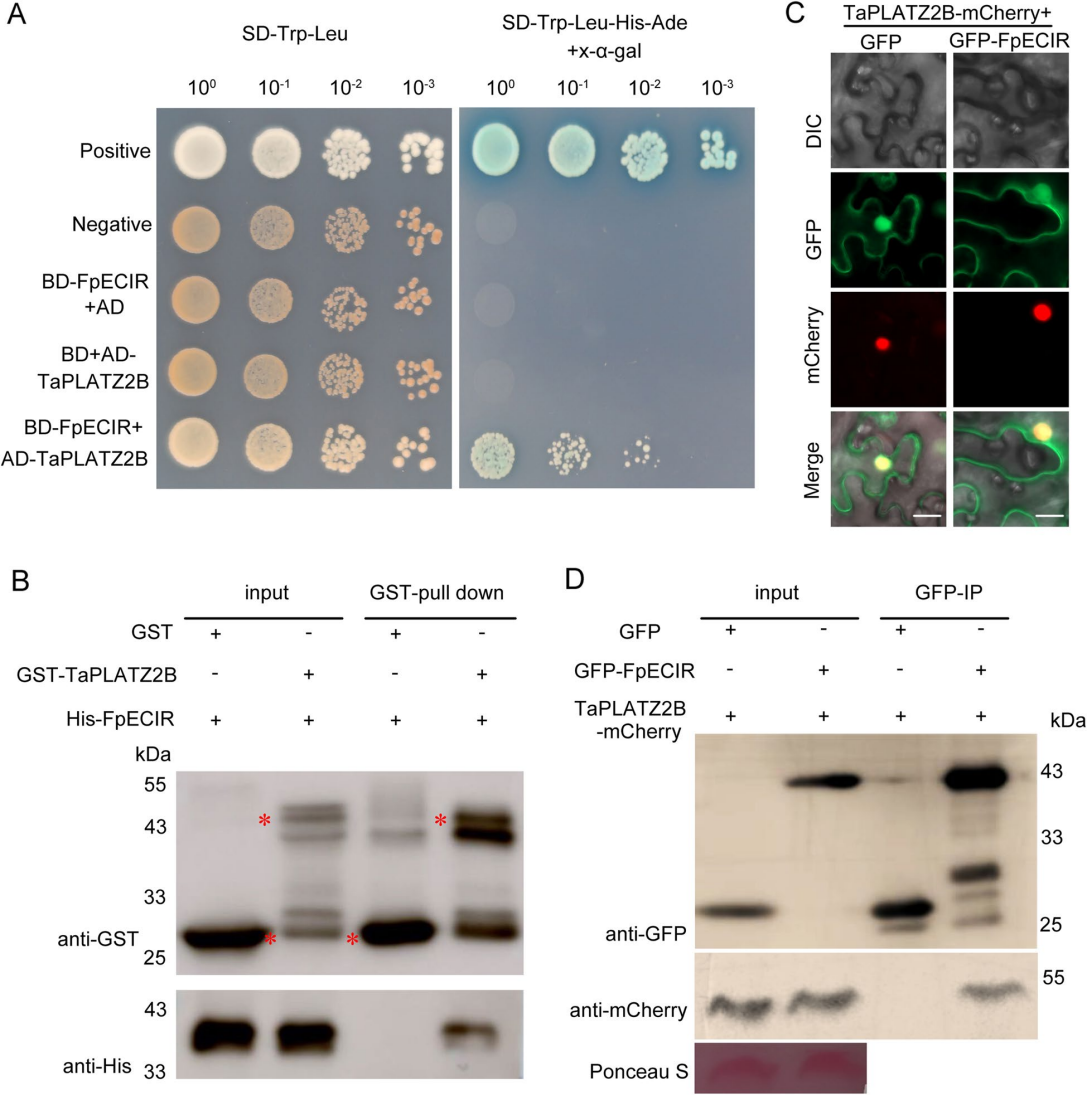
FpECIR interacts with the transcription factor TaPLATZ2B. **A** Verification the interaction between FpECIR and TaPLATZ2B by yeast two hybrid assays. Co-transformed BD-FpECIR with empty vector AD or BD with AD-TaPLATZ2B into Y2H strain, both of them can grow in SD-Trp-Leu medium but not SD-Leu-Trp-His-Ade plates, suggesting FpECIR and TaPLATZ2B did not have self-activation ability. Whereas, co-transformed BD-FpECIR with AD-TaPLATZ2B into Y2H strain can grow on the SD-Trp-Leu and SD-Leu-Trp-His-Ade plates when the cells were serially diluted 10-, 100-, and 1000-fold from 1 × 10^6^ cells/mL initially, suggesting they can interact with each other. **B** GST pull-down assays were performed to validate the interaction between FpECIR and TaPLATZ2B. His-FpECIR and GST-TaPLATZ2B were expressed in DE3 strains, respectively. Anti-Glutathione MagBeads were used for inoculation with the protein complexes. Western blot following immunoblot analysis were used for detecting the corresponding proteins with His and GST antibodies. **C** Co-localization GFP or GFP-FpECIR and TaPLATZ2B-mCherry in *N.benthamiana* leaves. After co-expressing *GFP *or *GFP*-*FpECIR* with *TaPLATZ2B*-mCherry in *N.benthamiana* leaves, the infiltrated leaves were observed by confocal microscopy 36 h post-infiltration (hpi). GFP-FpECIR localized in nucleus and plasma membrane, while TaPLATZ2B-mCherry localized in the nucleus alone. The green and red fluorescence signals colocalized in the nucleus. **D** Co-IP assays for the interaction of FpECIR with TaPLATZ2B. Total proteins were extracted using NP-40 lysis buffer from *N.* *benthamiana* leaves co-expressing GFP-FpECIR and TaPLATZ2B-mCherry. Proteins complexes were inoculated with anti-GFP agarose beads and the corresponding proteins was detected by western blot following immunoblot analysis with GFP and mCherry antibodies

Since TaPLATZ2B was originally identified using a yeast two hybrid assay, it was selected for subsequent experiments. The full-length *TaPLATZ2B* gene was cloned and constructed as prey, and its interaction with FpECIR was confirmed by yeast two-hybrid assay (Fig. 4A). Additionally, a pull-down assay was performed to verify their interaction in vitro. Recombinant His-*FpECIR*, GST-*TaPLATZ2B* and GST were expressed and purified in the *Escherichia coli* strain DE3 respectively. GST or GST-TaPLATZ2B was co-incubated with His-FpECIR using glutathione high-capacity magnetic agarose beads. Western blot analysis showed that His-FpECIR was enriched only in the beads bound to GST-TaPLATZ2B but not in those bound to GST (Fig. 4B). To assess the interaction in planta, *GFP* or *GFP-FpECIR* was co-expressed with *TaPLATZ2B*-mCherry in *N. benthamiana* leaves via *Agrobacterium*-mediated transient expression. At 36 h post-infiltration, confocal microscopy revealed overlapping green and red fluorescence signals in the nucleus, suggesting colocalization and potential interaction between FpECIR and TaPLATZ2B (Fig. 4C). To further verify this interaction in vivo, Co-immunoprecipitation (Co-IP) assays were performed using proteins that were extracted from *N. benthamiana* leaves co-expressing GFP or GFP-*FpECIR* with *TaPLATZ2B*-mCherry. Western blot analysis showed that TaPLATZ2B-mCherry was detected in complexes co-expressed with GFP-FpECIR but not with GFP alone (Fig. 4D). These data suggest that FpECIR interacts with TaPLATZ2B both in vivo and in vitro.

### Key interaction sites were identified of FpECIR with TaPLATZ2B

Previous work showed that the N-terminal and C-terminal of FpECIR cannot suppress INF1 triggered cell death. To determine whether the two mutants could still interact with TaPLATZ2B, yeast two-hybrid assays were performed using FpECIR-N and FpECIR-C as bait and TaPLATZ2B as prey. Results showed that only FpECIR-N retained the ability to interact with TaPLATZ2B, whereas FpECIR-C lost the ability (Figure S4).

**Fig. 5:**
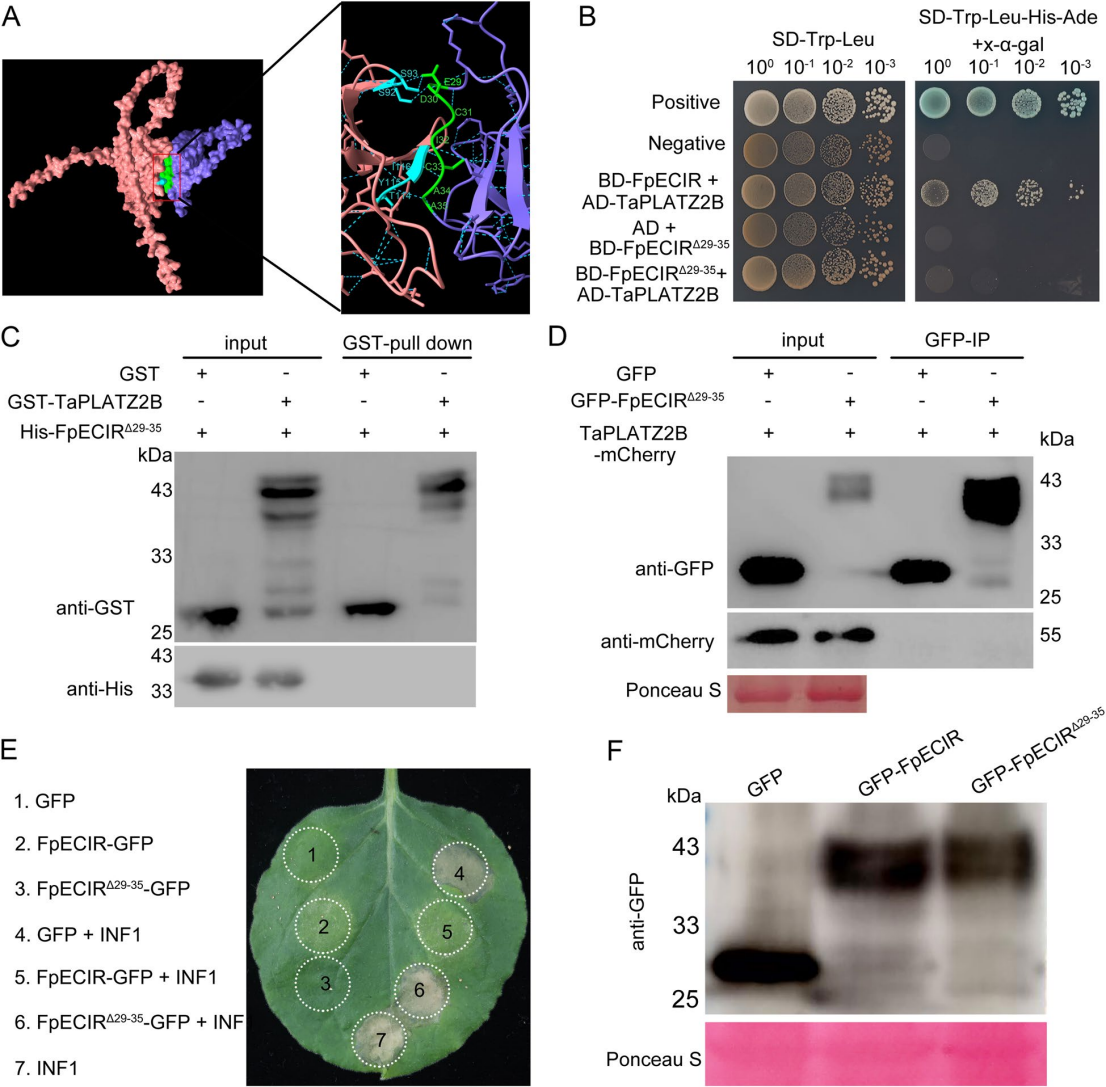
Residues 29 to 35 of FpECIR play key role for its function. **A** Structural model of FpECIR with TaPLATZ2B interaction (left). Residues 29 to 35 of FpECIR (the green part) were predicted to interact with different residues of TaPLATZ2B, Glu29 and Asp30 with Ser92 and Ser93, Cys33 with Tyr115 and Ile116, and Ala35 with Thr114 (right). **B** Validation the interaction of FpECIR^Δ29–35^ with TaPLATZ2B yeast two hybrids. BD-FpECIR^Δ29–35^ did not have self-activation ability, and lost the ability to interacted with TaPLATZ2B. **C**, **D** GST pull down and Co-IP were carried out to examine the interaction between BD-FpECIR^Δ29–35^ and TaPLATZ2B. the two experiments proved BD-FpECIR^Δ29–35^ cannot interact with TaPLATZ2B. **E** Transient expression *FpECIR*^Δ29–35^-GFP in *N.* *benthamiana* leaves 24 h prior to infiltration of the INF1-carrying strain GV3101, full-length FpECIR-GFP still can suppress INF1-triggered cell death, whereas FpECIR^Δ29–35^-GFP cannot. **F** Western blot and immunoblot analysis of the protein expressing GFP and FpECIR-GFP and FpECIR^Δ29–35^-GFP in *N.* *benthamiana* leaves

To further identify the specific sites of FpECIR that interact with TaPLATZ2B, the structures and interaction model of the two proteins were predicted using Alphafold v3 (Fig. 5A, left). Based on the predicted hydrogen bonds formed between FpECIR and TaPLATZ2B, residues 29 to 35 of FpECIR were predicted to binding with different residues of TaPLATZ2B, Glu29 binding with Ser92, Asp30 binding with Ser93, Cys33 binding with Tyr115 and Ile116, and Ala35 binding with Thr114 (Fig. 5A, right). Therefore, whether the mutant FpECIR^Δ29–35^ could interact with TaPLATZ2B was tested using yeast two-hybrid, GST pull-down and Co-IP assays. The results showed that FpECIR^Δ29–35^ was unable to interact with TaPLATZ2B (Fig. 5B-D). To confirm this result, two mutants were generated: TaPLATZ2BM1 (92^S^93^S^ to 92^A^93^A^) and TaPLATZ2BM2 (114–116^TYI^ to 114–116^AAA^). These mutants were used as prey in yeast two hybrid assays, and the results revealed that TaPLATZ2BM1 but not TaPLATZ2BM2 could interact with FpECIR (Figure S5), which supports the predicted interaction sites. In addition, transient expression of FpECIR^Δ29–35^ in *N. benthamiana* leaves failed to suppress INF1 induced cell death unlike the full-length FpECIR (Fig. 5E). Expression of FpECIR^Δ29–35^ was confirmed by Western blot analysis (Fig. 5F). These data suggested that the N-terminal is the functional domain of FpECIR and that residues 29–35 are crucial for its interaction with TaPLATZ2B and for suppressing plant immunity.

### *TaPLATZ2B* positively regulates the expression of wheat defense-related genes against FCR

**Fig. 6:**
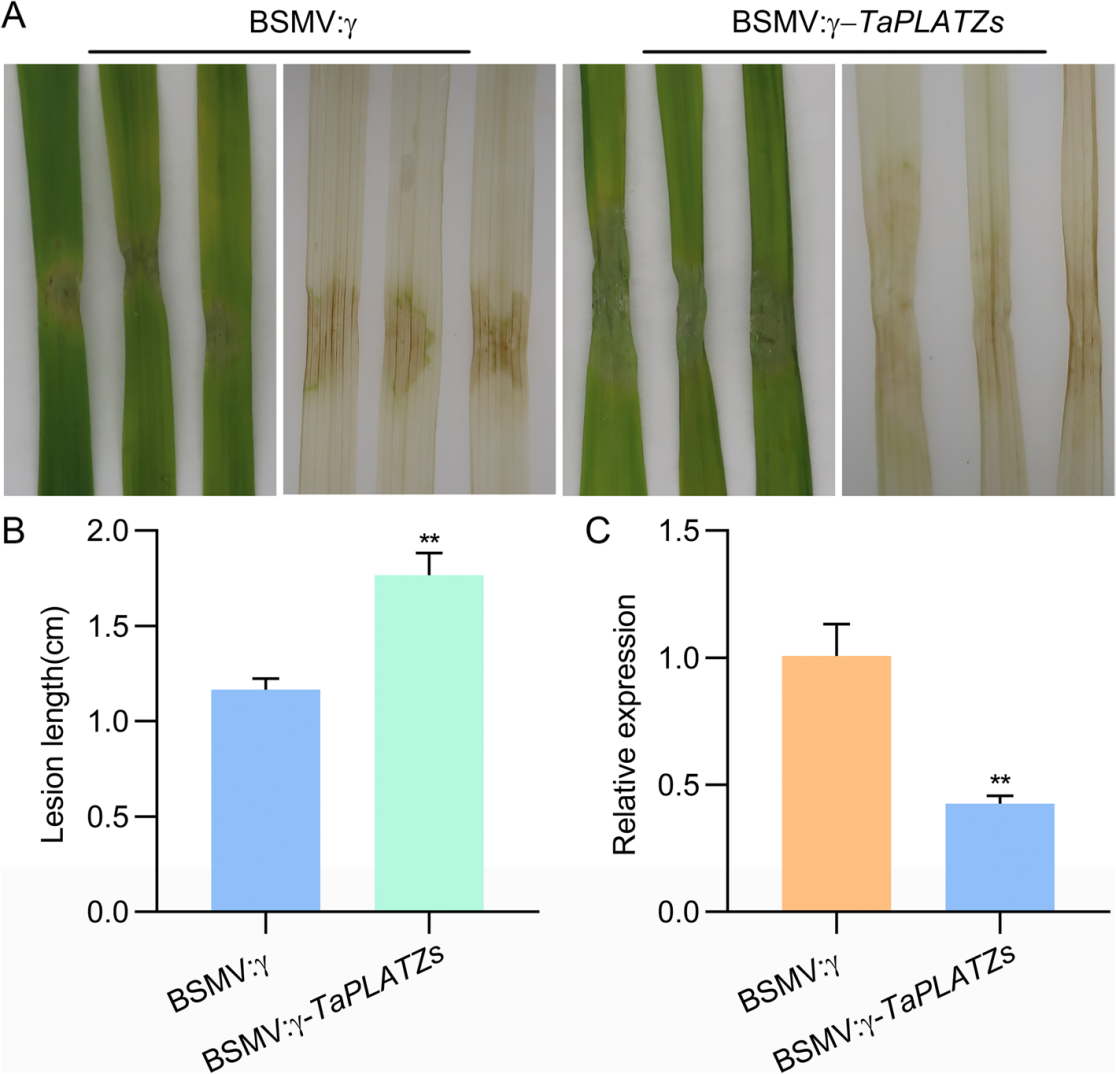
Silencing *TaPLATZs* increase the susceptivity of wheat to *F. pseudograminearum* infection. **A** Disease symptoms of wheat leaves treating with virus sap and inoculated with wild type *F. pseudograminearum*. The wheat leaves were inoculated with BSMV-γ or γ: *TaPLATZs* virus sap, 12 dpi, the upper second or third leaves were inoculated with wild type *F. pseudograminearum*, 36 hpi, the mycelial plugs were removed and 72 hpi, the corresponding leaves were photographed after decolorated in the alcohol. **B** The lesion length was measured and estimated from three independent experiments. Asterisks indicate significantly differences based on the Student's *t*-test analysis, *P* < 0.01. **C** Relative expression levels of *TaPLATZ2B* genes in BSMV-γ or γ: *TaPLATZs* virus sap treated leaves 12 dpi were examined by qRT-PCR. Asterisks indicate significantly differences based on the Student's *t*-test analysis, *P* < 0.01. Experiments were repeated three times

To evaluate the role of *TaPLATZ2B* in wheat resistance to *F. pseudograminearum* infection, barley stripe mosaic virus (BSMV)-mediated virus-induced gene silencing (VIGS) assay was performed to transiently silence all three *TaPLATZ2B* alleles in wheat simultaneously. The fragment selected for silencing was inserted into the γ vector (γ:*TaPLATZs*) and transformed into *Agrobacterium tumefaciens* strain GV3101. *N. benthamiana* leaves at the four-leaf stage were inoculated with a mixed GV3101: suspension containing BSMV: α, β, and γ with *TaPDS* or *TaPLATZs*. At 12 dpi, the second upper leaf was harvested and ground in extraction buffer to obtain virus sap, which was mechanically inoculated onto wheat leaves at three-leaf stage. Two weeks later, the indicator plants treated with BSMV: γ: *TaPDS* treated leaves showed obvious bleaching (Figure S6A), the second and third leaves above the treated leaves were selected for transcriptional analysis, and to evaluate wheat disease resistance. qRT-PCR results showed that the relative expression level of *TaPLATZ2B* was significantly reduced by approximately 30%−60%. Inoculation of the *F. pseudograminearum* on wheat leaves indicated that *TaPLATZs*-silenced plants displayed more severe symptoms and longer lesions than control plants treated with BSMV:γ (Fig. 6A-C). In addition, *TaPLATZs* silenced wheat appeared thinner and weaker under the same growth conditions compared to the BSMV: γ inoculated wheats (Figure S6B). These results indicate that silencing *TaPLATZs* not only affects wheat development but also increases wheat susceptibility towards *F. pseudograminearum* infection, which suggesting that *TaPLATZ* positively regulates wheat resistance to *F. pseudograminearum*.

### *FpECIR* contributes to the full virulence of *F. pseudograminearum*

**Fig. 7:**
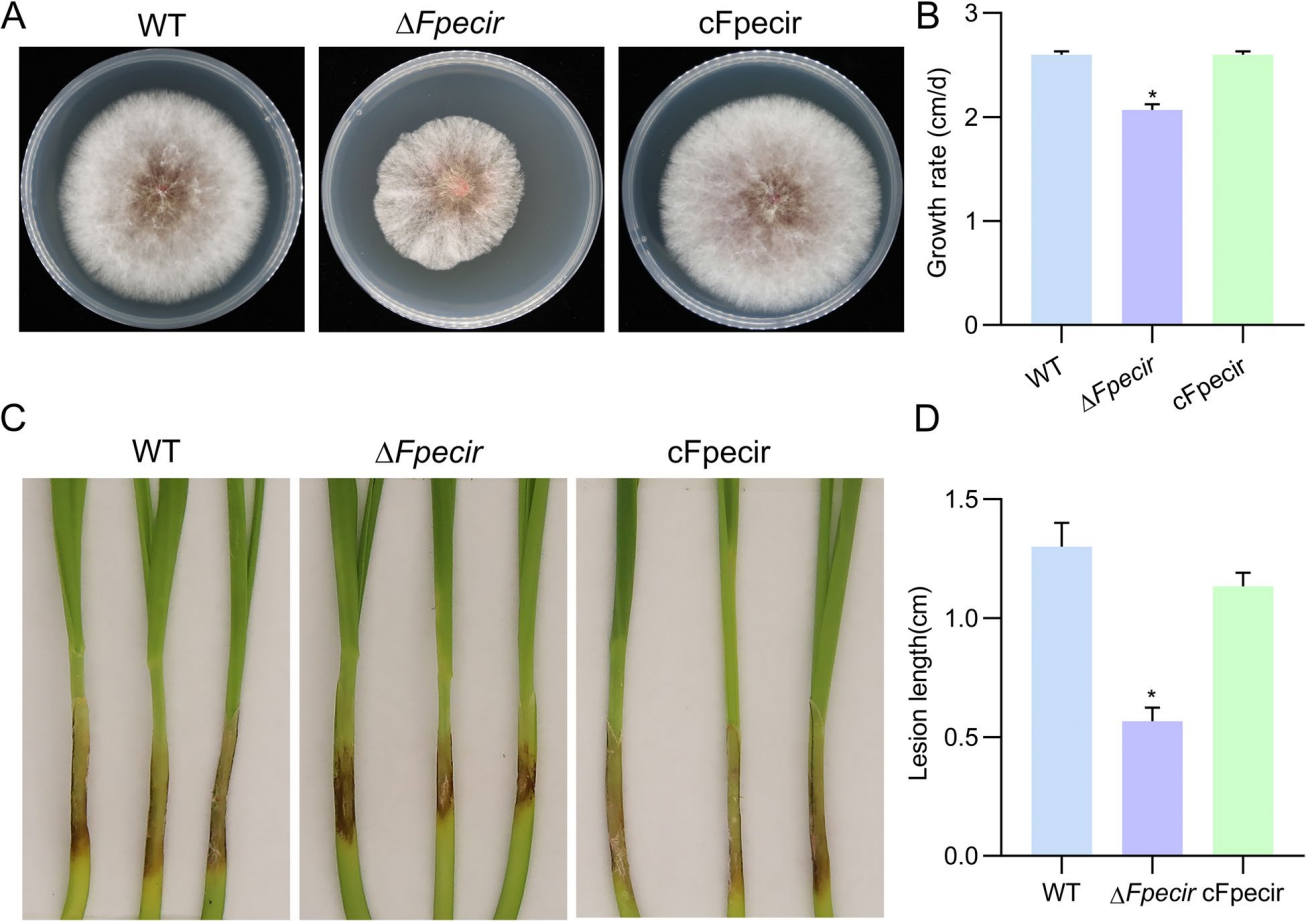
*FpECIR* contributes to the full virulence of *F. pseudograminearum* to wheat. **A** Images of wild-type, deletion mutant Δ*Fpecir* and the complement cFpecir strains cultured on PDA plates at 25 °C for 3 d in the dark. **B** Growth rate of the three strains in PDA plates. Data represent the means of three independent replicates. Asterisk indicates differences based on Student’s *t*-tests, *P* < 0.05, experiments repeated three times. **C** Images of symptoms on wheat seedlings were photographed at 72 hpi. The wheat cultivar AK58 was inoculated with wild type, deletion mutant Δ*Fpecir* and the complement cFpecir strains. **D** The lesion lengths were measured from the wheat seedlings inoculated with different strains at 72 hpi. Asterisk indicates differences based on the Student's *t*-test analysis, *P* < 0.05, experiments repeated three times

To further confirm the role of *FpECIR* in *F. pseudograminearum* pathogenicity, deletion mutants Δ*Fpecir* were generated by replacing the gene with the hygromycin resistance gene, and the mutants were verified by PCR (Figure S7). Complemented transformants of Δ*Fpecir*, termed cFpecir, were also constructed. The growth rate of Δ*Fpecir* was slower than that of the wild type (WT) and cFpecir, but the deletion mutants did not affect spore production (Fig. 7A, B and S8). However, inoculation assays on wheat coleoptiles showed that compared with the WT or cFpecir, the Δ*Fpecir* mutants induced less severe symptoms and shorter lesion length (Fig. 7C, D). These data implied that *FpECIR* is necessary for the virulence of *F. pseudograminearum* in wheat coleoptiles.

### The influence of deletion mutant Δ*Fpecir* on the transcriptional levels of wheat

**Fig. 8:**
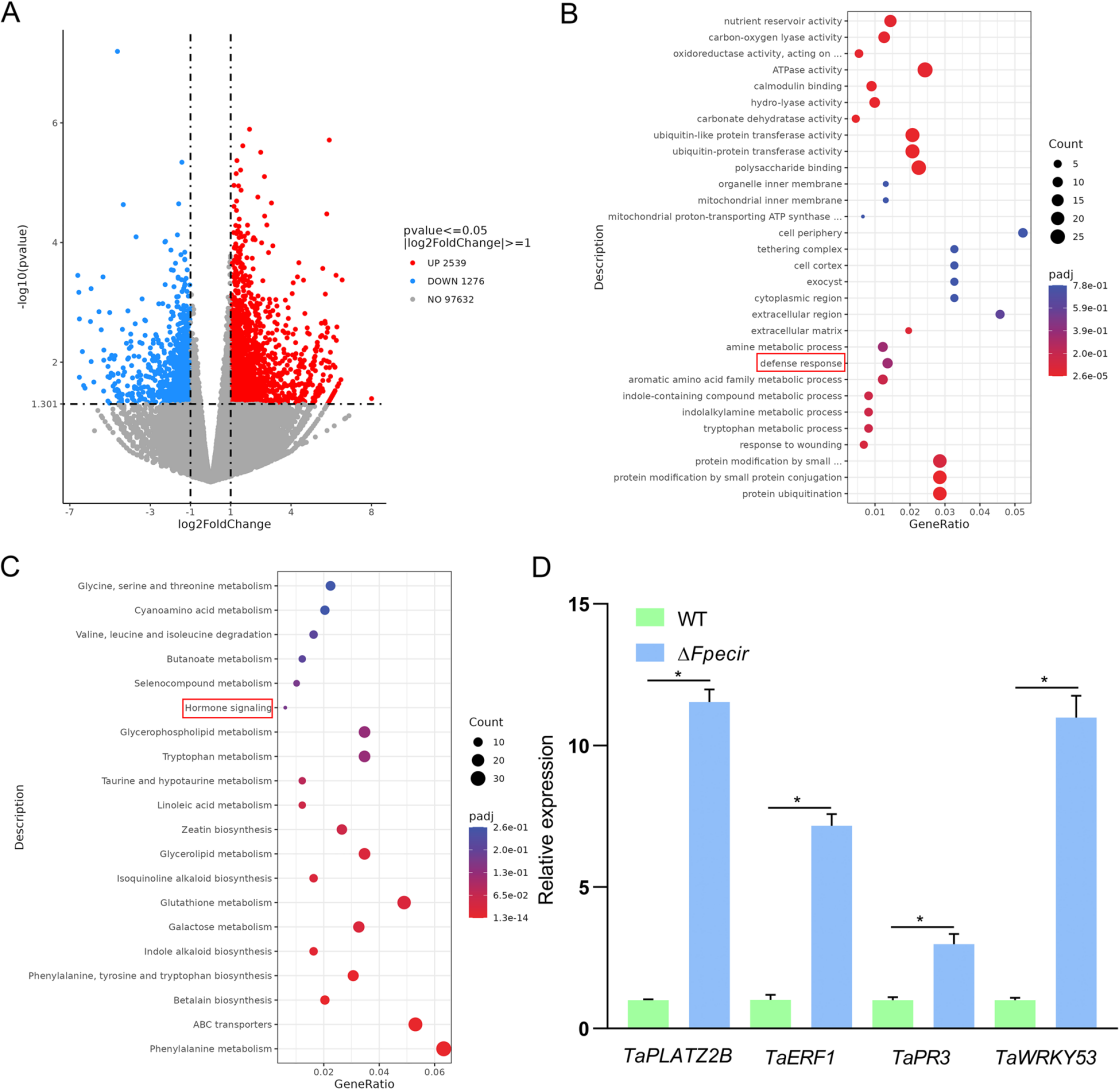
RNA-Seq the genes expression in wheat coleoptiles inoculated with wild type and Δ*Fpecir* strains. **A** Wheat coleoptoles inoculated with wild type and Δ*Fpecir* strains were collected following RNA-Sequencing. Differentially expressed proteins were analyzed by volcano plot based on the transcriptome analysis. Blue plots represent the genes with upregulated expression, red plots represent the genes with downregulated expression and the gray part represents the genes’ expression without change in the wheat coleoptiles inoculated with Δ*Fpecir* strains compared that inoculated with wild type. **B** GO enrichment analysis of the 2539 upregulated genes. The 30 most significant terms were selected to draw a scatter plot for presentation based on the results of the GO enrichment analysis foe the upregulated genes. **C** KEGG enrichment analysis of the 2539 upregulated genes. The 20 most significant KEGG pathways were selected to draw a scatter plot for presentation based on the KEGG enrichment results. **D** Validation the expression levels of *TaPLATZ2B* and ethylene signaling pathway related genes (*TaERF1*, *TaPR3*, and *TaWRKY53*) using qRT-PCR. Different RNAs were extracted from wheat seedlings inoculated with wild type and Δ*Fpecir* strains. All experiments repeat three times, asterisk indicates significantly differences based on the Student's *t*-test analysis, *P* < 0.05, experiments were repeated three times

The Δ*Fpecir* significantly affected the pathogenicity of *F. pseudograminearum*. RNA sequencing of wheat coleoptiles infected with the Δ*Fpecir* and wild type strains was performed to analyze the transcriptome-wide expression differences caused by the deletion of *FpECIR*. The results showed that 2,539 genes were up-regulated and 1,276 genes were down-regulated in coleoptiles infected with the deletion mutants Δ*Fpecir* (Fig. 8A, Table S3). Gene ontology (GO) enrichment analysis of the transcriptome data revealed that defense response was presented among the top 30 terms, indicating that FpECIR may suppress plant immune responses (Fig. 8B). This is consistent with the finding that FpECIR suppresses INF1-triggered immunity (Fig. 2A). Additionally, KEGG pathway enrichment analysis revealed that FpECIR not only plays a key role in various metabolic and biosynthetic pathways but may also be involved in hormone signaling pathways (Fig. 8C). These results indicate that deletion of *FpECIR* could enhance the disease resistance in wheat against *F. pseudograminearum* infection to a certain extent, which may be caused by influencing the various metabolic or biosynthetic process, or even the hormone signaling pathway.

**Fig. 9:**
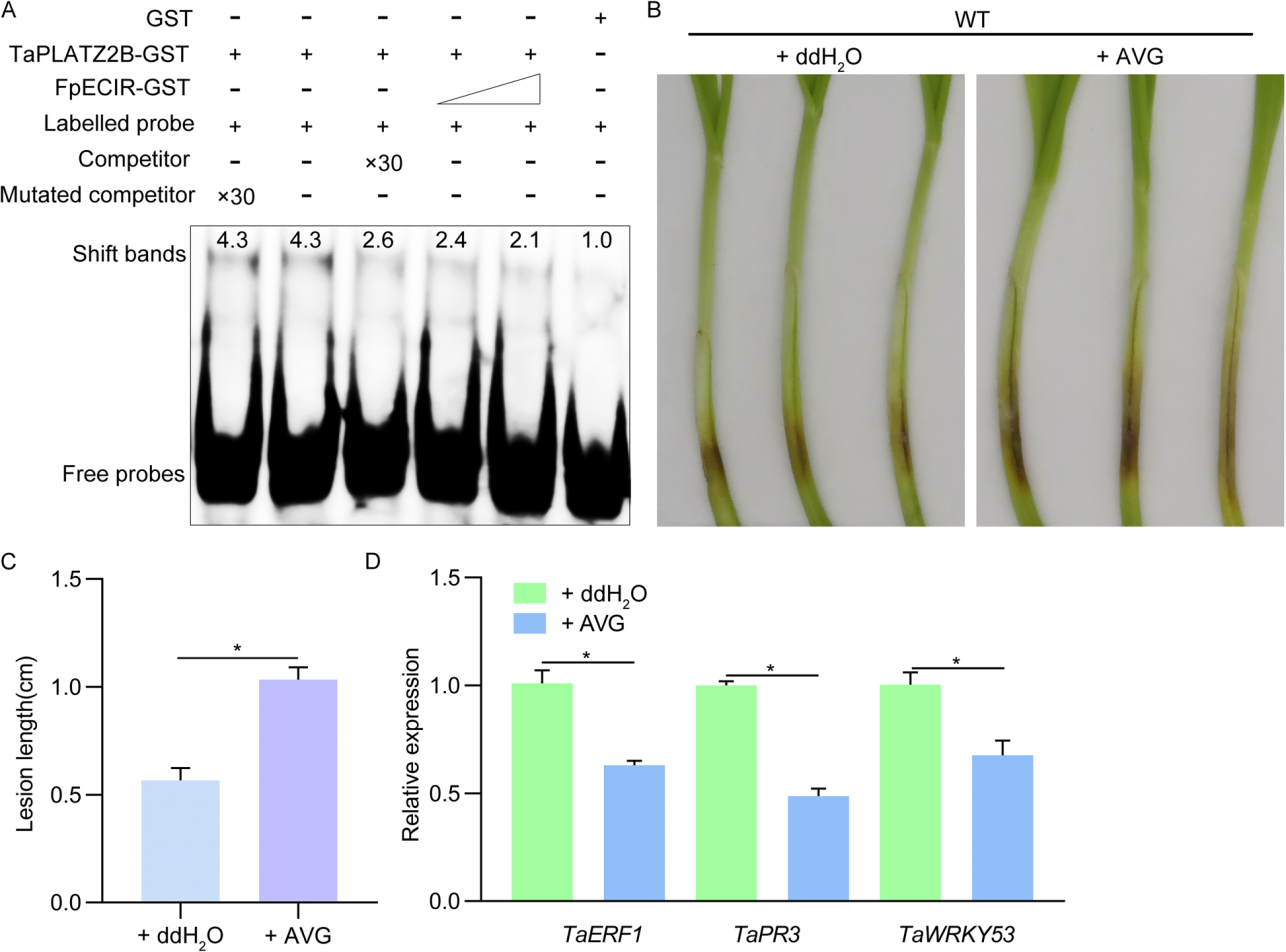
Ethylene signaling pathway positively regulates wheat defense against *F. pseudograminearum* infection. **A** EMSA analysis of the effect of FpECIR on the binding of TaPLATZ2B with *TaERF020L* promoter. Unlabelled and mutated probes were used as competitor. ‘ + ’ and ‘-’ indicate the presence and absence, respectively. mutated probes did not affect the TaPLATZ2B binding to *TaERF020L* promoter, while with the addition of the FpECIR protein into the reaction system, the binding ability of TaPLATZ2B to *TaERF020L* promoter decreased significantly. Gray values of the shift bands were analyzed by ImageJ, and the numbers represented the binding ability of TaPLATZ2B with probe. **B** Wheat seedlings were pretreated with either AVG (+ AVG) or ddH₂O (control, -AVG) for 24 h prior to inoculation with the wild-type strain. Images of symptoms on wheat seedlings were photographed at 48 hpi. **C** The lesion lengths were measured from the corresponding wheat seedlings. Asterisk indicates differences based on the Student's *t*-test analysis, *P* < 0.05, experiments repeated three times. **D** Validation the expression levels of *TaERF1*, *TaPR3* and *TaWRKY53* using qRT-PCR. Different RNAs were extracted from *F. pseudograminearum* inoculated wheat seedlings which were treated with AVG or not. All experiments repeat three times, asterisk indicates significantly differences based on the Student's *t*-test analysis, *P* < 0.05, experiments were repeated three times

To investigate whether FpECIR influences the expression of *TaPLATZ2B*, which was not detected in the transcriptome data, qRT-PCR assays were performed to measure the relative expression level of *TaPLATZ2B* in wheat coleoptiles inoculated with Δ*Fpecir* or wild type. The results demonstrated that the presence of FpECIR suppressed the expression of *TaPLATZ2B* (Fig. 8D). Previous reports have showen that *TaPLATZ2B* is involved in the response of plants to abiotic stress through the abscisic acid (ABA), gibberellin (GA) and ethylene pathways (Zhang et al. 2018). Transcriptome data showed that many ethylene signaling associated genes were significantly upregulated (Table S3). To confirm this, qRT-PCR was performed on three representative ethylene pathway related genes, *TaPR3* (AB029936), *TaERF1* (EF583940) and *TaWRKY53* (EF368364.1) (Fig. 8D). Additionally, two genes from other hormone signaling pathways were analyzed: *TaPR1* (AF384143) belonging to salicylic acid (SA) signaling pathway and *TaPR6* (EU293132) associated with jasmonic acid (JA) signaling pathway (Figure S9). The results showed that the transcription levels of all five genes were significantly up-regulated in coleoptiles infected with deletion mutants Δ*Fpecir*. Together with the transcriptome data, these results indicate that FpECIR inhibits the expression of *TaPLATZ2B*, thereby affecting the expression of genes related to ethylene and other hormone signaling pathways, and consequently influencing the function of *TaPLATZ2B*. In addition, the the EMSA assay was performed to confirm whether FpECIR influences the DNA-binding activity of TaPLATZ2B. Here we choose an ethylene response factor (LOC123066087, TaERF020L), of which expression level was higher in the wheat seedlings infected by the Δ*Fpecir* strain than the wild type according to the RNA-Seq data (Table S3). A 42 bp probe from the promoter region of *TaERF020L* was designed and tagged by biotin. The GST, FpECIR-GST and TaPLATZ2B-GST proteins were expressed and purified in the *Escherichia coli* strain DE3 respectively. The results showed that TaPLATZ2B directly bound to the biotin-labeled probe from *TaERF020L* promoter region, while the binding ability decreased following the addition of FpECIR protein into the reaction system (Fig. 9A). These results give the evidences that FpECIR affects the function of *TaPLATZ2B* by inhibiting its transcription of it and ethylene signaling pathways.

To further examine the function of ethylene in wheat immunity, Aminoethoxyvinylglycine (AVG) hydrochloride, one ethylene biosynthesis inhibitor, has been used to suppress the expression of genes related to ethylene signaling pathway. Wheat seedings were treated with 1 mM AVG and the coleoptiles were inoculated with mycelial plugs (wild type) 24 h later. Inoculation assays showed that coleoptiles treated with AVG had more severe disease symptoms and longer lesion length (Fig. 9B, C). In addition, qRT-PCR was carried out to test the expressions of *TaPR3*, *TaERF1* and *TaWRKY53* in coleoptiles treated with AVG or not. The results showed that, regardless of whether the plants were inoculated with *F. pseudograminearum*, the expression levels of these three genes in AVG-treated wheat seedlings were significantly decreased compared to those in ddH_2_O treated coleoptiles (Figure S10, Fig. 9D). These findings proved that ethylene is important for wheat against the infection of *F. pseudograminearum*.

## Discussion

PAMP triggered immunity (PTI) and effector triggered immunity (ETI) constitute the two branches of plant immune system that protect plants from pathogen infection during the long-term host-microbe interactions (Jones and Dangl 2006). Fungal pathogens are known to secrete hundreds of small proteins, termed “effectors”, into the extracellular or intracellular space in order to manipulate the plant immune system and facilitate infection (Tariqjaveed et al. 2021; Wang et al. 2020). Numerous secreted proteins with unknown functions have been identified through transcriptome analysis in *F. graminearum*, *Colletotrichum higginsianum* and *Phytophora sojae* (Brown et al. 2012; Kleemann et al. 2012; Wang et al. 2011), but the function of most of these effectors remains unclear. At the same time, no functional domains were predicted in FpECIR using the SMART online tool, and its role during the *F. pseudograminearum*-wheat interaction stage had not been characterized. However, proteins lacking identifiable domains have been shown to play important roles in facilitating pathogen infection. For example, the orphan protein Osp24, which lacks any known domains, can suppress BAX- or INF1- triggered cell death, and modulates plant immunity by binding more strongly to TaSnRK1α than to TaFROG, thereby accelerating TaSnRK1 degradation and enhancing the pathogen virulence (Jiang et al. 2020). In *F. pseudograminearum*, FpCDP1 and Fp00392 are two apoplastic effectors capable of inducing cell death in *N. benthamiana* leaves and are required for the pathogen’s full virulence on wheat (Liu et al. 2025; Yang et al. 2025a). In the present study, transient expression of FpECIR in tobacco leaves effectively suppressed INF1-triggered cell death, and deletion of *FpECIR* significantly reduced the pathogenicity of *F. pseudograminearum*. These findings demonstrate that FpECIR functions as a virulence-associated effector and plays an important role in suppressing host immune responses during infection.

TaPLATZ2B, identified as the interaction target of FpECIR by yeast two hybrid screening, was further investigated to elucidate its role in plant immunity and development, as well as the molecular mechanism by which FpECIR modulates host defense responses. In this study, the interaction between FpECIR and TaPLATZ2B was validated using multiple approaches, and both proteins were found to colocalize in the nucleus. We speculate that FpECIR may exert its function through TaPLATZ2B. Previous studies have shown that homologs of TaPLATZ2B in different plants participate in responses to biotic and abiotic stresses and affect plant development. In maize, the RGF1-induced protein RIF1, a member of the PLATZ family, is a key regulator of root meristem size. Silencing *ritf1*/*2*/*3* in maize resulted in a reduced meristem and slower root growth, whereas overexpression of *RITF1* in wild type plants led to an enlarged root meristem size and enhanced the superoxide (O^2-^) accumulation (Yamada et al. 2020). In apple, identified *PLATZ* genes exhibited distinct expression patterns and tissue-specific characteristics. Drought or ABA treatments affected their expression, and transcriptome analysis indicated that *PLATZ* genes respond to drought stress by regulating the ABA signaling pathway. Consistent with these findings, *PLATZ* genes in ginkgo and *Populus* species also display pronounced, tissue-specific expression patterns, as revealed by RNA-Seq (Han et al. 2022; Sun et al. 2022; Wang et al. 2024). In rice, grain size and number are critical determinants of yield. The PLATZ transcription factor GL6 regulates grain length and weight by controlling cell proliferation, while simultaneously exerting a negative effect on grain number (Wang et al. 2019). In this study, silencing *TaPLATZ2B* and its alleles resulted in delayed wheat development, similar to the functions of *PLATZ* genes reported in maize and rise mentioned above. In Tartary buckwheat, *FtPLATZ3* and *FtPLATZ4* have been shown to regulate of grain size, whereas *FtPLATZ11* is involved in root development. The transcription levels of *FtPLATZ6* were induced by ABA, GA, and SA treatments, while other *FtPLATZ* genes exhibited different expression patterns in response to these hormones (Li et al. 2022). In the present study, qRT-PCR validated that *TaPLATZ2B* was upregulated in wheat coleoptiles infected with deletion mutants Δ*Fpecir* compared with the wild type. In addition, KEGG pathway enrichment analysis indicated that hormone signaling pathway were prominently involved in wheat responses to *F. pseudograminearum* infection. Silencing *TaPLATZ2B* and its allele lead wheat growth slower and more susceptible to *F. pseudograminearum* infection, these results reveled that the functions of *PLATZ* are conserved in different plants.

Reactive oxygen species (ROS) signaling responses to biotic and abiotic stresses by activating expression of defense genes. The ERFs were activated when plants facing different stresses that could enhance ROS production. In Arabidopsis, ERF6 can be phosphorylated and activated by MAPK3 to trigger the expression of ROS related genes, which enhances stress tolerance (Müller and Munné-bosch 2015; Sewelam et al. 2013). Here we choose an up-regulated gene *TaERF020L* based on the RNA-Seq data to confirm if its expression was affected by FpECIR. EMSA assays proved TaPLATZ2B can bind with the probe from *TaERF020L* promoter region, but binding ability of TaPLATZ2B with probe decreased following the addition of FpECIR. Furthermore, qRT-PCR assays confirmed that genes associated with ET signaling pathway were all upregulated in Δ*Fpecir* infected wheat seedlings. Previous studies have shown that *TaPLATZ5* contributes to wheat defense against *Blumeria graminis* f. sp. *tritici* (*Bgt*) infection (Song et al. 2022), combined with the result that TaPLATZ2B confers wheat resistance to *F. pseudograminearum*, which suggest that *TaPLATZ* may function as a wide-spectrum resistance gene in wheat. However, no studies to date have reported on the effects of effectors on the function of PLATZ transcription factors.

Phylogenetic tree and NCBI Blast results revealed that only a limited number of species harbor homologs genes of *FpECIR*. These homologs are distributed among plant pathogens, animal pathogens, biocontrol fungi and other fungi, suggesting that these genes may have undergone functional diversification during evolution. Multiple sequence alignment of the amino acid sequences encoded by these genes showed that the predicted N terminal internal repeats is highly conserved. AlphaFold 3 is a powerful tool for predicting protein structures, and protein–protein interactions and has been successfully applied to model complexes such as PvPGIP2-FpPG (Abramson et al. 2024a; Xiao et al. 2024). In this study, Alphafold v3 was employed to predict the three-dimensional (3D) structure of FpECIR. Structural modeling revealed that the two internal repeats of FpECIR adopt highly similar conformations, and functional analyses demonstrated that both repeats are required for FpECIR-mediated suppression of INF1-triggered cell death. Additionally, the interaction model between FpECIR and TaPLATZ2B revealed their potential binding sites. Deletion or mutation of these predicted sites in FpECIR caused the mutants to lose the ability to interact with TaPLATZ2B. These results demonstrate the reliability of the prediction outcomes.

In summary, the cytoplasmic effector FpECIR not only suppresses INF1-induced cell death, but also modulates wheat defense responses by inhibiting the expression of *TaPLATZ2B* and further downregulating hormone signaling related genes. Silencing *TaPLATZs* significantly impaired wheat development and resistance. Taken together with previous studies, these findings suggest that *TaPLATZ2B* represents a promising regulatory node for coordinating wheat disease resistance and yield-related traits.

## Conclusion

**Fig. 10:**
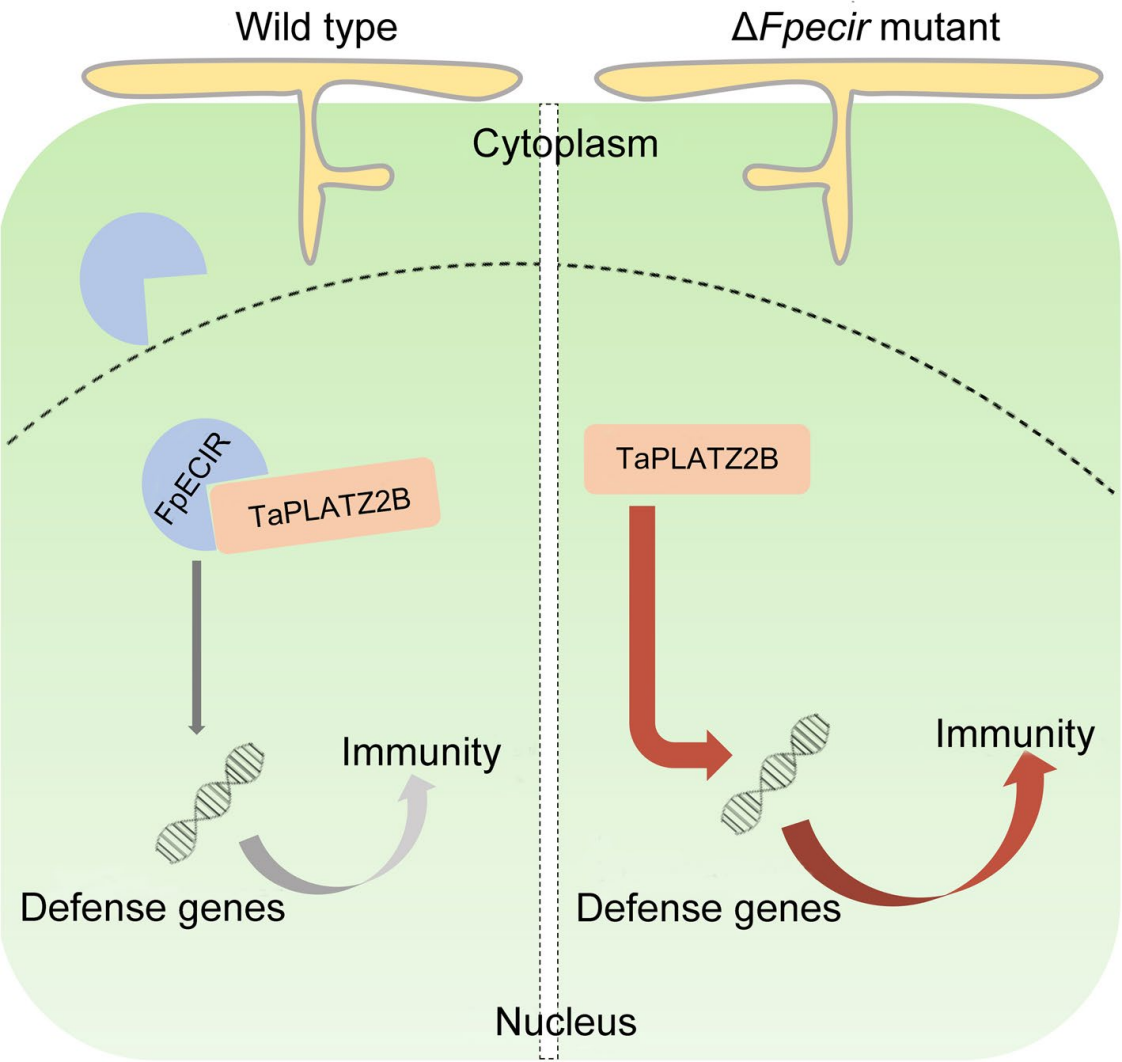
A schematic model of FpECIR regulates plant immunity by inhibiting the function of TaPLATZ2B. TaPLATZ2B is a transcription factor to regulate wheat defense response through ethylene signaling pathway. Relative transcription level of TaPLATZ2B was higher in the wheat seedlings infected by the deletion mutant strain Δ*Fpecir* than the wild type. In addition, FpECIR physically interacted with TaPLATZ2B and affected the binding ability of TaPLATZ2B with the promoter of ethylene response factor *TaERF020L*, and thus decreased wheat resistant to FCR

Overall, this study illustrates that FpECIR functions as a cytoplasmic effector from *F. pseudograminearum* that suppresses INF1-induced cell death. Multiple independent assays verified that FpECIR physically interacts with TaPLATZ2B. FpECIR contributes the virulence of *F. pseudograminearum* by inhibiting the expression of *TaPLATZ2B* and reducing its binding to the promoter regions of hormone signaling-related genes, consequently downregulating their expression. The N-terminal internal repeat is highly conserved and sequence integrity were necessary for the function of FpECIR. Notably, *TaPLATZ2B* acts as a positive regulator of wheat resistance against *F. pseudograminearum*, highlighting its importance as a host target manipulated by this effector (Fig. 10).

## Materials and methods

### Bioinformatics analysis

Signal peptide prediction was performed using SignalP-6.0 (https://services.healthtech.dtu.dk/services/SignalP-6.0/), transmembrane domain prediction using TMHMM-2.0 (http://www.cbs.dtu.dk/services/TMHMM/), and conserved domain prediction using SMART (https://smart.embl.de/). Protein structure and protein interaction models were predicted using AlphaFold 3 (Abramson et al. 2024b) and visualized with ChimeraX. Homologs screening of FpECIR was conducted using NCBI blast, and the phylogenetic tree was constructed with MEGA-X and visualized using iTOL v7 (https://itol.embl.de/).

### Strains and culture conditions

The wild type *F*. *pseudograminearum* strain WZ-8A was cultured on potato dextrose agar (PDA) medium at 25℃. *Escherichia coli* (*E. coli*) strain DH5α and DE3, used for plasmid construction and prokaryotic expression respectively, were cultured in LB medium at 37 °C. *Agrobacterium tumefaciens* (*A. tumefaciens*) strain GV3101 was cultured in LB medium at 28 °C and used for transient expression.

### Plasmids construction

The *FpECIR* genes with or without signal peptide were cloned from cDNA of *F*. *pseudograminearum* strain WZ-8A and the *TaPLATZ2B* gene was cloned from cDNA of wheat cultivar AK58 with gene specific primers (Supplementary Table S1). Amplified *FpECIR* fragments were inserted into pGBKT7 (BD), pBin-GFP2, PVX-GFP and pET32a-His vectors. Amplified *TaPLATZ2B* fragments were inserted into pGADT7 (AD), pBin-mCherry and pGEX4T-2-GST vectors. All recombinant vectors were constructed using the In-Fusion Cloning Kit (Vazyme Biotech, Nanjing, China) and transformed into DH5α for positive clone screening, which was subsequently verified by sequencing. In addition, small *TaPLATZ2B* fragments for gene silencing were cloned from BD-*FpECIR* and inserted into BSMV: γ vector with the T4 DNA Ligase Kit (NEB-China, Beijing, China). Primers used here were listed in table S1.

### Verification the function of FpECIR signal peptide

The predicted signal peptide (SP) of FpECIR was cloned and inserted into the pSUC2 vector (pSUC2-FpECIRSP) and the reconstructed plasmid was transformed into the yeast strain YTK12 (Oh et al. 2009), colonies were grown on SD-Trp medium. Meanwhile, pSUC2-Avr1bSP and the empty vector pSUC2 were also transformed into YTK12 as positive and negative controls, respectively (Dou et al. 2008). A functional SP is able to make colorless 2, 3, 5-triphenyltetrazolium chloride (TTC) to insoluble red-colored 1, 3, 5-triphenylformazan (TPF) due to the activity of the invertase enzyme encoded in pSUC2 plasmid.

To figure out whether FpECIR was secreted into the intracellular or extracellular space, its native promoter together with the coding sequence was cloned and inserted into the pKNT-*mCherry*−3× *NLS* vector. The generated construct was transformed into wild type *F. pseudograminearum* protoplasts and the resulting transformants were used for infection assays on wheat seedlings (Jiang et al. 2020). mCherry fluorescence signals were observed through microscope at two days post inoculation (dpi). Primers used here were listed in table S1.

### *Agrobacterium tumefaciens* mediated transient expression

The GV3101 strain harboring the specific plasmid was cultured in LB medium with certain antibiotics at 28 °C, 220 rpm overnight. *Agrobacterium tumefaciens* cells were collected by centrifugation at 5000 rpm for 3 min at room temperature and resuspended in infiltration buffer (10 mmol/L MgCl_2_, 1 mmol/L MES pH 5.6, and 200 μmol/L acetosyringone). *Nicotiana benthamiana* plants were grown in a greenhouse under a 16/8 h light/dark cycle and 4–5-week-old leaves were selected for infiltration.

For suppression of INF1-triggered cell death in effector experiments, PVX-GFP was introduced as backbone vector to construct PVX-*FpECIR*-*GFP* and other plasmids containing *FpECIR* mutants. These plasmids were then transformed into GV3101 strains. At 24 h post-inoculation (hpi) of *N. benthamiana* leaves with agrobacterium carrying *FpECIR-GFP *fusion gene, the same sites were infiltrated with agrobacterium expressing INF1, and leaf images were captured seven days later. Meanwhile, the functions of *FpECIR*-related mutants were evaluated using the same method.

### Yeast two hybrid assays

The *FpECIR* gene was inserted into the pGBKT7 plasmid to generate the bait construct (BD-*FpECIR*) for screening a cDNA library constructed from RNA extracted from wheat coleoptiles infected with *F. pseudograminearum*. SD-Trp-Leu-His-Ade medium were used to select the potential interacting proteins. Candidate colonies were sequenced using T7 primer and the resulting sequences were identified by BLAST analysis in EnsemblPlants (https://plants.ensembl.org/index.html). The full length *TaPLATZ2B* gene was cloned from AK58 cDNA and inserted into the pGADT7 plasmid to get the prey construct (AD-*TaPLATZ2B*). BD-*FpECIR* and AD-*TaPLATZ2B* were co-transformed into the yeast strain Y2HGold, growth conditions were assessed on SD-Trp-Leu medium and SD-Trp-Leu-His-Ade medium were used for assessing the interaction between the two proteins. BD-*FpECIR* with the empty vector AD or AD-*TaPLATZ2B* with the empty vector BD were co-transformed into Y2HGold, and SD-Trp-Leu-His-Ade medium were used to test whether the two proteins exhibited self-activation ability. Interactions of FpECIR or TaPLATZ2B related mutants with other protein were verified using the same method.

### Co-immunoprecipitation (Co-IP) and GST-Pull down experiments

Co-immunoprecipitation (Co-IP) assays were carried out following the method reported before (Yang et al. 2018), *GFP* or *GFP*-*FpECIR* and *TaPLATZ2B*-mCherry fusion genes were co-expressed in four-week-old *N. benthamiana* leaves and these leaves were collected 36 h after infiltration, and grand in liquid nitrogen using a TissueMaster™ High-Throughput tissue homogenizer at 50 Hz, for 60 s and repeat this step 3–6 times. Proteins were extracted using the NP-40 lysis buffer (50 mM Tris-HCl (pH7.4), 150 mM NaCl, 1% NP-40 and 1 mM phenylmethylsulfonyl fluoride (PMSF)). The proteins extracts were centrifuged at 14 000 rpm for 10 min at 4 °C and this step was repeated twice. Supernatants were incubated with 20 μL Anti-GFP Affinity Beads 4FF (Smart-Lifesciences, Changzhou city, China) at 4 °C for 4 h with gentle rotation, then the beads were collected and washed five times with washing buffer (50 mM Tris-HCl (pH 7.4), 100 mM NaCl). The proteins attached to the beads were washed and boiled in water for 8 min in SDS-PAGE sample loading buffer. Protein complexes were detected by SDS-PAGE followed by immunoblotting with specific antibodies.

For GST pull-down assays, GST, *TaPLATZ2B*-GST and *FpECIR*-His fusion genes were expressed in DE3 strains were cultured in LB medium at 37 °C, 220 rpm overnight. Subsequently, the cultures were diluted (1:100) in fresh LB medium and incubated at 37 °C until reaching an OD_600_ of approximately 0.6. Protein expression was induced by 0.5 mM isopropylthio-β-galactoside (IPTG) at 20 °C, 150 rpm for 12 h. Cells were harvested by high-speed centrifugation and resuspended in PBS buffer (10 mM Na_2_HPO_4_, 2 mM NaH_2_PO_4_, 135 mM NaCl, 4.7 mM KCl, pH7.4). The cells were disrupted using an ultrasonic cell crusher to solubilize the recombinant proteins in the buffer. Pull-down assays were performed by incubating FpECIR-His with GST or TaPLATZ2B-GST using Glutathione MagBeads GST (Thermo Fisher Scientific, Cleveland, OH, USA), and subsequent steps were conducted as described for Co-IP assay.

### Generation of *FpECIR* deletion mutants

A split marker approach was used to construct the gene replacement of *FpECIR*, preparation of the *F. pseudograminearum* protoplasts and PEG-mediated transformation were performed according to previously described procedures (Liu et al. 2025). Upstream and downstream flanking fragments of *FpECIR* were amplified from WZ-8A genomic DNA using specific primers and fused with the hygromycin resistance gene (*Hph*) by overlapping PCR. A third round PCR was carried out to amplify two fragments, upstream of *FpECIR* with two third of upstream *Hph*, downstream of *FpECIR* with two third of downstream *Hph*. Both fragments were transformed into *F. pseudograminearum* protoplasts. Hygromycin was added to the medium at a final concentration of 50 μg·mL^-1^ to select positive transformants. PCR assays were carried out to test if the *FpECIR* was replaced by *Hph*. In addition, fragments containing *FpECIR* gene and its promoter region were amplified using specific primers from genomic DNA and inserted into the pKNT-GFP vector, which carries a neomycin (*NEO*) resistance gene cassette as a selection marker. The reconstructed vectors were transformed into protoplasts of *FpECIR* deletion mutants and screen on PDA medium containing 50 μg·mL^-1^ G418. Primers used here were listed in table S1.

### Wheat coleoptiles infection assays

Wheat coleoptile infection assays were carried out according to the method described previously (Ma et al. 2022). Briefly, wheat seedlings were grown on greenhouse for 4 days, and coleoptiles were inoculated with 5 mm diameter mycelial plugs. At 24 hpi, fungal discs were removed once clear symptoms were observed on coleoptiles infected by wild type or mutant strains. At 72 hpi, lesion lengths of wheat coleoptiles were measured and photographed. All experiments were repeated at least three times and statistical analyses were using one-way ANOVA. Samples for RNA sequencing were collected at 72 hpi from wheat seedlings infected with wild type and Δ*Fpecir* strains.

To investigate the effect of ethylene on wheat against *F. pseudograminearum* infection, 1 mM Aminoethoxyvinylglycine hydrochloride (AVG) (MACKLIN, Shanghai, China), one kind of ethylene biosynthesis inhibitor, was sprayed onto the surface of wheat seedlings (Kumari et al. 2025), while some wheat seedlings were sprayed with ddH_2_O as control. 24 h later, the different treated coleoptiles were inoculated with 3 mm diameter mycelial plugs. Fungal discs were removed at 24 hpi and lesion lengths of wheat coleoptiles were measured and photographed at 48 hpi.

### Quantitative real-time polymerase chain reaction (qRT-PCR) assays

Total RNA from wheat coleoptiles infected by different strains were extracted using the RNA isolater Total RNA Extraction Reagent (Vazyme, Nanjing, China) according to the product manual instructions. cDNA was synthesized using HiScript IV RT SuperMix for qPCR (+ gDNA wiper) (Vazyme, Nanjing, China) following the instructions. qPCR was performed using the ChamQ Blue Universal SYBR qPCR Master Mix (Vazyme, Nanjing, China) and reaction system were prepared following the manufacture’ instructions. *FpEF1α* and *TaEF1α* were used the internal control genes to normalize the data and relative expression levels were calculated using the 2^-ΔΔCt^ method and statistical analyses were using t-tests (Jiang et al. 2024; Livak and Schmittgen 2001; Wang et al. 2022). Primers used for qRT-PCR were listed in the supplementary table S1.

### BSMV-mediated gene silencing in wheat

To explore gene function in wheat, a barley stripe mosaic virus (BSMV) mediated gene silencing system was employed (Yuan et al. 2011). Conserved small fragments of *TaPLATZ2B* and its two alleles was cloned from cDNA derived from wheat cultivar AK58 and inserted into the BSMV:γ (pCa-γbLIC) vector. The reconstructed plasmid was transformed into *A. tumefaciens* strain GV3101. Four-leaf-stage *N*. *benthamiana* plants were used for inoculation with *A. tumefaciens* strains carrying the BSMV:α, BSMV:β and either BSMV: γ (negative control), γ-*PDS* (positive control) or γ-*TaPLATZs*. Strains containing different vectors were adjusted to equal cell densities (OD_600_≈0.7). At 12 dpi, *N*. *benthamiana* leaves from differently treated were collected and ground in virus exreaction buffer, The buffer was prepared by first mixing 11.5 mL of 0.2 M Na_2_HPO_4_ (pH = 5.8), 1 mL 0.2 M NaH_2_PO_4_ (pH = 5.8), and 0.5% (w/v) sodium sulfite solution to make 10 mM PBS, and then combining 5 mL of this PBS with 20 mL sodium sulfite solution and 95 mL ddH_2_O. Commonly, 0.2 g leaves was ground in 10 mL buffer. Inoculation of wheat with leaf sap was performed as described in the study (Fan et al. 2019). Wheat leaves inoculated with BSMV: γ served as negative control and γ-*PDS* as positive control. About 12 dpi, γ-*PDS* treated leaves showed an obvious bleaching, qRT-PCR was used to verify the transcription levels of *TaPLATZ2B* in BSMV: γ or γ-*TaPLATZs* treated wheat leaves. To evaluate wheat resistance against *F. pseudograminearum*, 3 mm diameter mycelial plugs were inoculated on the second or third leaves above the treated leaves. the mycelial plugs were removed after 36 hpi, and the inoculated leaved were photographed at 72 hpi and the lesion length were measured at the same time. The collected leaves will be decolorated in the alcohol for clearly observe the symptoms. Primers used here were listed in table S1.

### Electrophoretic Mobility Shift Assay (EMSA)

The GST, FpECIR-GST and TaPLATZ2B-GST proteins were got as the method described in GST pull-down mention above. The EMSA assay were carried out according to the product manual instructions (Beyotime, Shanghai, China). The *TaERF020L* promoter region was predicted and selected as the method described in the papers (Song et al. 2022), the biotinylated probe contained the canonical CTATT motif (a 42-bp *TaERF020L* promoter fragment) was synthesized as DNA probe, the unlabeled and mutated probes were used as competitors. The gray values of shift bands were measured by ImageJ. Primers used here were listed in table S1.

## Supplementary Information

Supplementary Material 1.

Supplementary Material 2.

Supplementary Material 3.

Supplementary Material 4.

## Data Availability

All data generated or analyzed during this study are included in the published article and its supplementary information files.

## Abbreviations

FCR: Fusarium Crown Rot
DON: Deoxynivalenol
ROS: Reactive oxygen species
QTLs: Quantitative trait locis
WT: Wiled type
PAMP: Pathogen-associated molecular pattern
TFs: Transcription factors
PLATZ: Plant AT-rich sequence- and zinc-binding
FpECIR: *F. pseudograminearum* Effector with two Internal Repeats
BSMV: Barley stripe mosaic virus
ORF: Open reading frame
IR: Internal repeat
TTC: 2,3,5-Triphenyltetrazolium
TPF: 1,3,5-Triphenylformazan
GO: Gene ontology
JA: Jasmonic acid
ABA: Abscisic acid
GA: Gibberellin
PTI: PAMP triggered immunity
ETI: Effector triggered immunity
Avr: Avirulence
PDA: Potato dextrose agar
BD: PGBKT7
AD: PGADT7
SP: Signal peptide
dpi: Days post inoculation
hpi: Hours post-inoculation
Co-IP: Co-immunoprecipitation
PMSF: Phenylmethylsulfonyl fluoride
IPTG: Isopropylthio-β-galactoside
NEO: Neomycin
Hph: Hygromycin
qRT-PCR: Quantitative real-time polymerase chain reaction
EMSA: Electrophoretic Mobility Shift Assay
AVG: Aminoethoxyvinylglycine
