# Supplementary material for: Effector FpECIR from Fusarium pseudograminearum targets wheat ethylene signaling pathway to suppress plant immunity
Source: Stress Biol. 2026 Jul 2;6(1):47. doi: 10.1007/s44154-026-00311-7 (PMC13328696; doi:10.1007/s44154-026-00311-7)
Supplement: Supplementary file 1 — Supplementary Material 1. [file 44154_2026_311_MOESM1_ESM.docx]

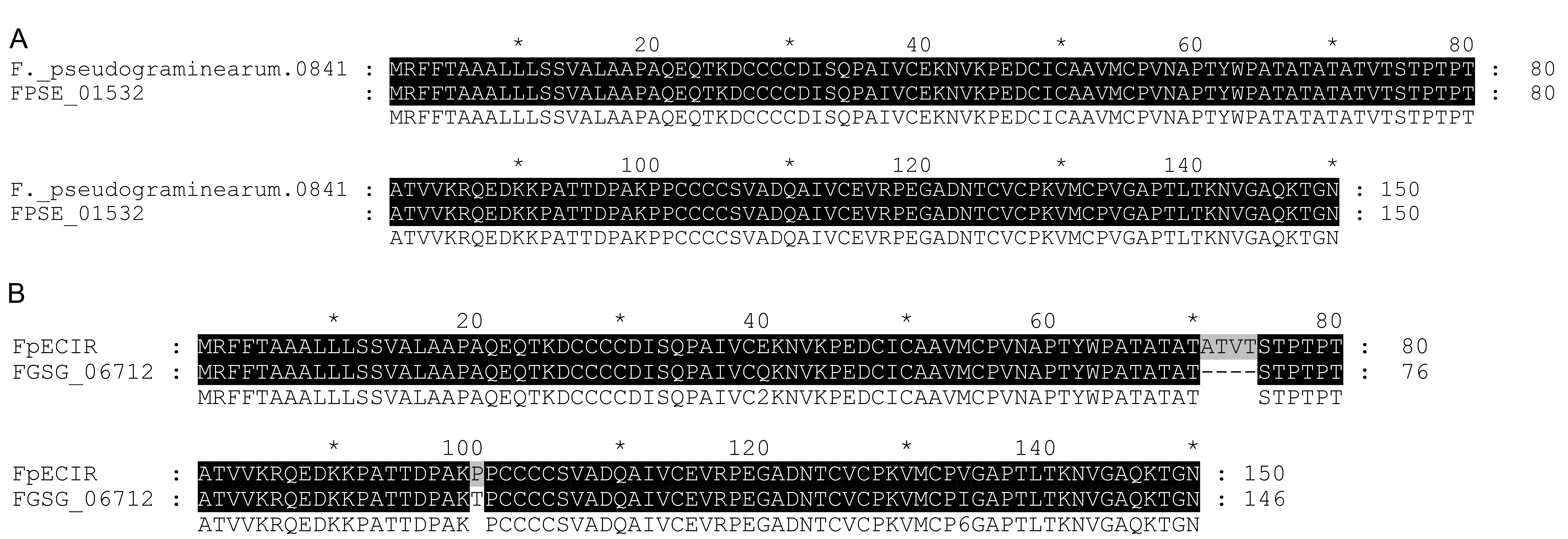


Figure S1. Sequences alignment of FpECIR in different strains. (A) sequences alignment of FpECIR from *F. pseudograminearum* 08419 (WZ-8A) or FPSE_01532 (*CS3096*) in different *F. pseudograminearum* strains. (B) Animo acid sequence alignment of FpECIR with its homologs in *F. graminearum* PH-1.


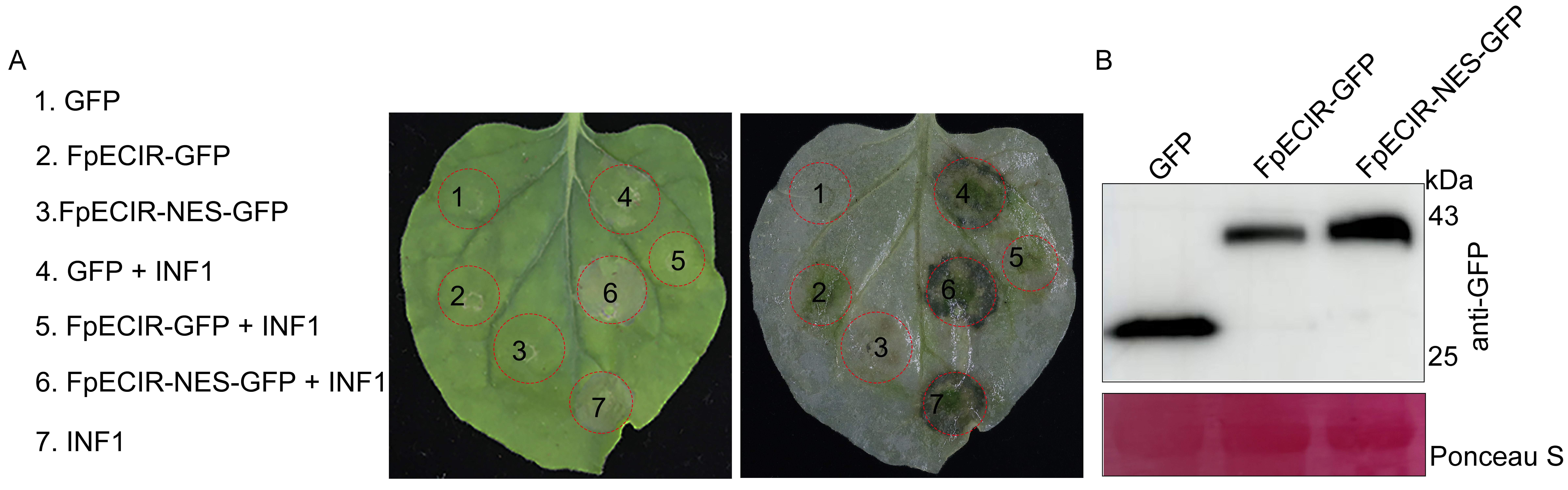


Figure S2. *FpECIR*-NES cannot suppress INF1 induced cell death. (A)Transient expression GFP, *FpECIR*-GFP and *FpECIR*-NES-GFP in *N. benthamiana* leaves 24 h prior to infiltration of the INF1-carrying strain GV3101, expressing GFP alone or fusion genes GFP, *FpECIR*-GFP or *FpECIR*-NES-GFP cannot induce cell death, while unlike *FpECIR*, expressing GFP and *FpECIR*-NES-GFP both cannot suppress INF1 induced cell death. Middle picture presents schematic drawing of the infiltration sites. The right picture represents the infiltrated leaf decolored by ethanol. (B) Western blot was used to examine the GFP and GFP-FpECIR proteins.


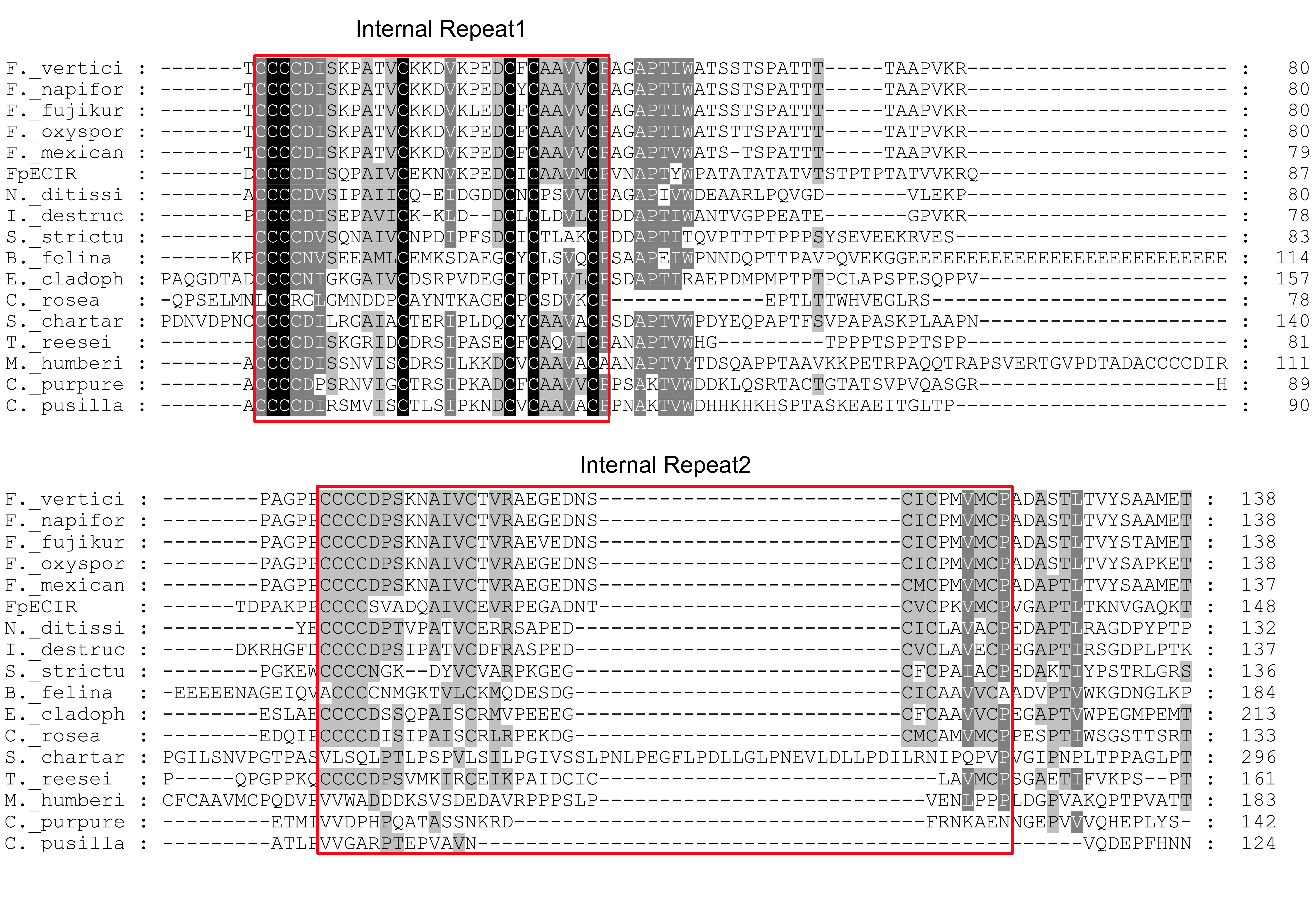


Figure S3. Multiple alignment of Sequences from different species. Red boxes represents two internal repeats predicted by SMART.


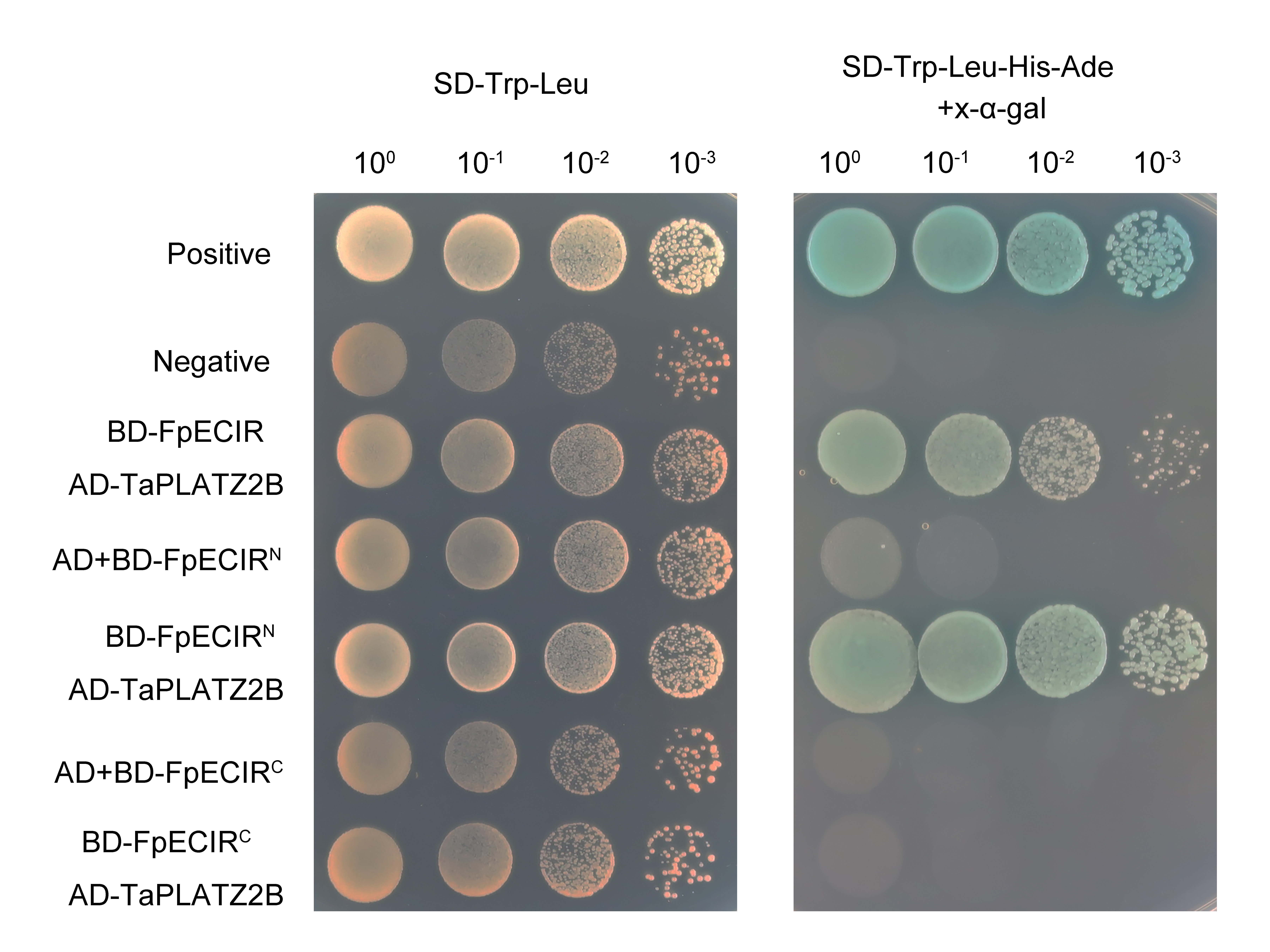


Figure S4. Verification the interaction between FpECIR^N^ or FpECIR^C^ and TaPLATZ2B by yeast two hybrid assays. Co-transformed BD-FpECIR^N^ with empty vector AD or AD-TaPLATZ2B into Y2H strain, both of them can grow in SD-Trp-Leu medium and SD-Leu-Trp-His-Ade plates, suggesting BD-FpECIR^N^ did not have self-activation ability and still can interacted with TaPLATZ2B. Whereas, BD-FpECIR^C^ did not have self-activation ability but also lost the ability to interact with TaPLATZ2B.


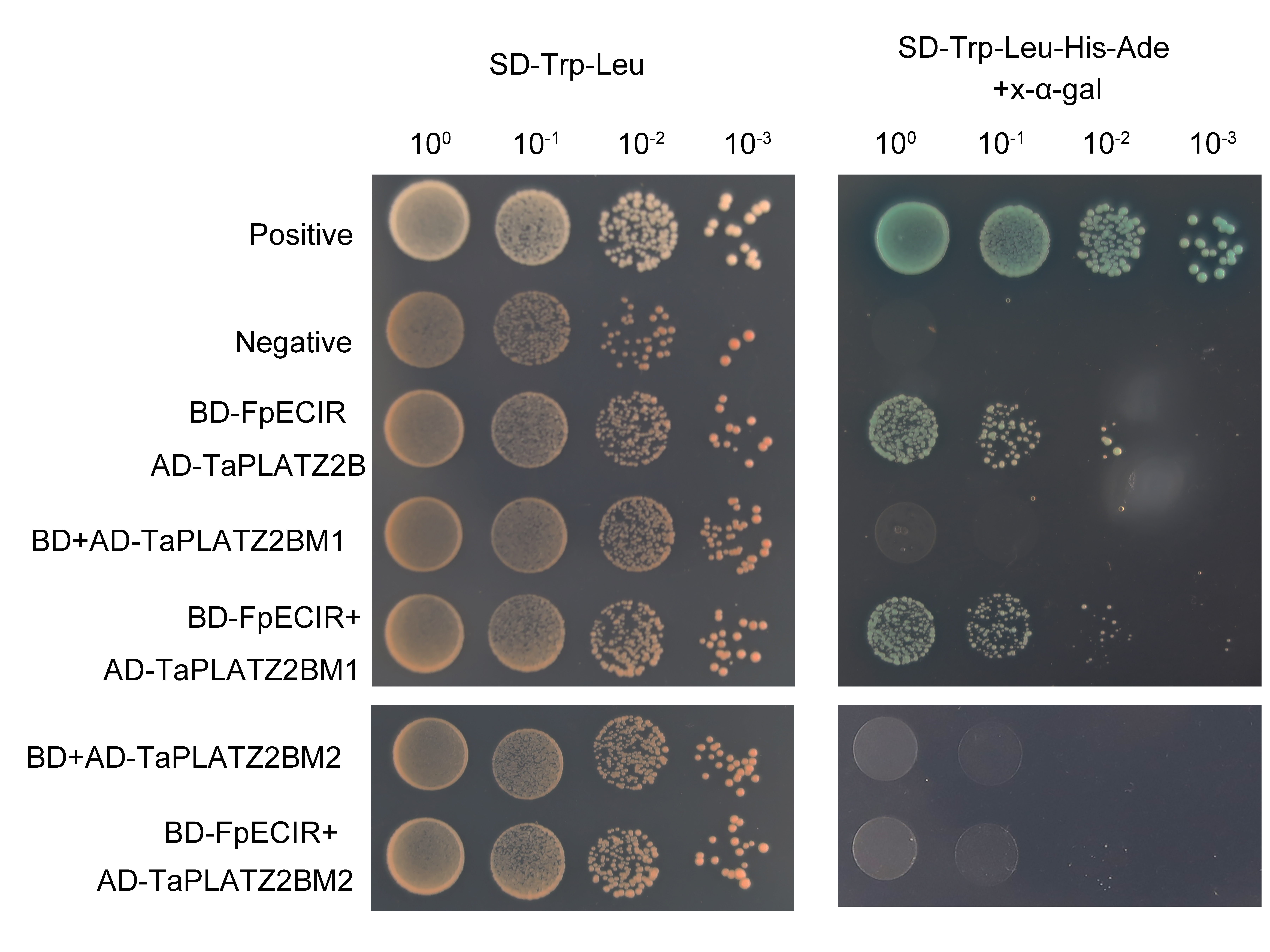


Figure S5. Verification the interaction between FpECIR and TaPLATZ2B mutants by yeast two hybrid assays. Co-transformed BD or BD-FpECIR with empty vector AD-TaPLATZ2BM1 into Y2H strain, both of them can grow in SD-Trp-Leu medium and SD-Leu-Trp-His-Ade plates, suggesting AD-TaPLATZ2BM1 did not have self-activation ability and still can interacted with BD-FpECIR. Whereas, Co-transformed BD or BD-FpECIR with empty vector AD-TaPLATZ2BM2 into Y2H strain, both of them can grow in SD-Trp-Leu medium but not SD-Leu-Trp-His-Ade plates, suggesting AD-TaPLATZ2BM2 did not have self-activation ability but also lost the ability to interact with BD-FpECIR.


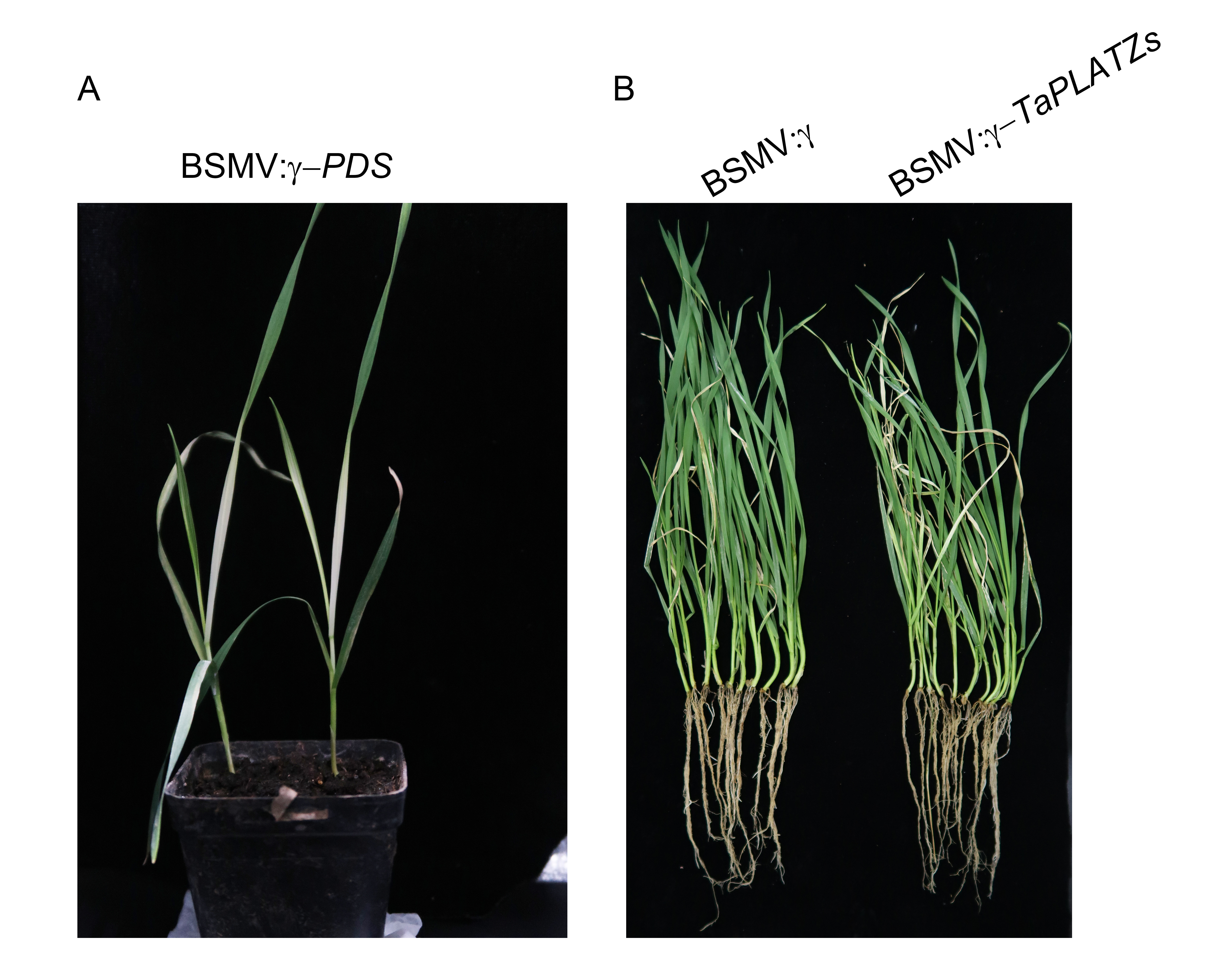


Figure S6. Silencing *TaPLATZ* may affect the development of wheat. (A) The control wheat leaves inoculated with BSMV: γ:*PDS* virus sap 12 dpi showed obvious bleaching. (B) The growth conditions of *TaPLATZ* silencing wheat.


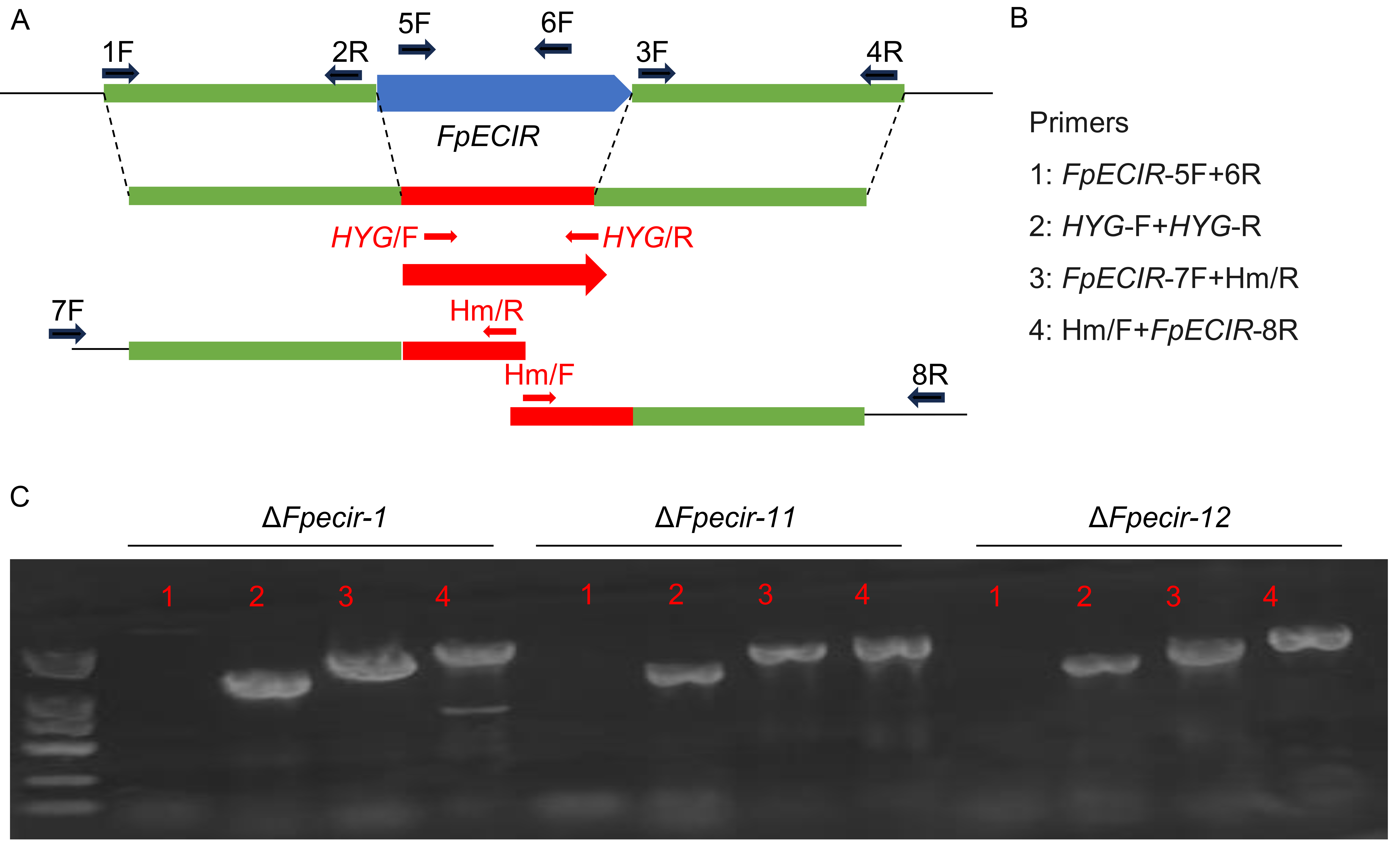


Figure S7. Schematic model of the *FpECIR* knockout and primers used for validation the transformants.


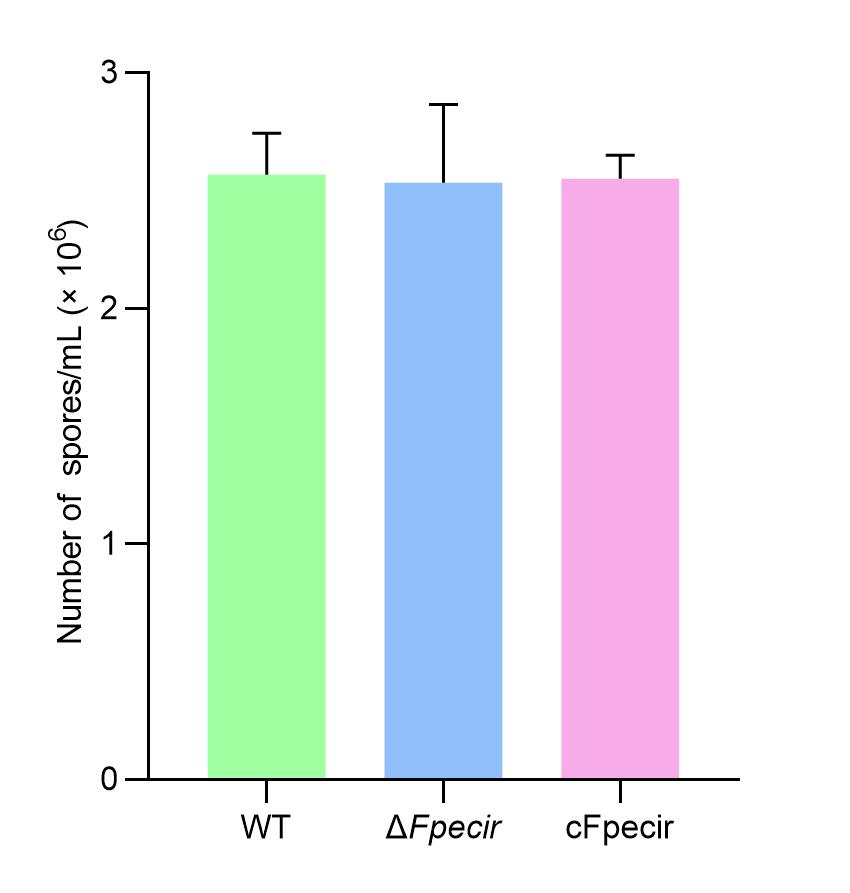


Figure S8. Spore production of different strains. Spore production of wild-type, deletion mutant Δ*Fpecir* and the complement cFpecir strains cultured on CMC medium. Data represent the means of three independent replicates. Data analysis was based on Student’s *t*-tests.


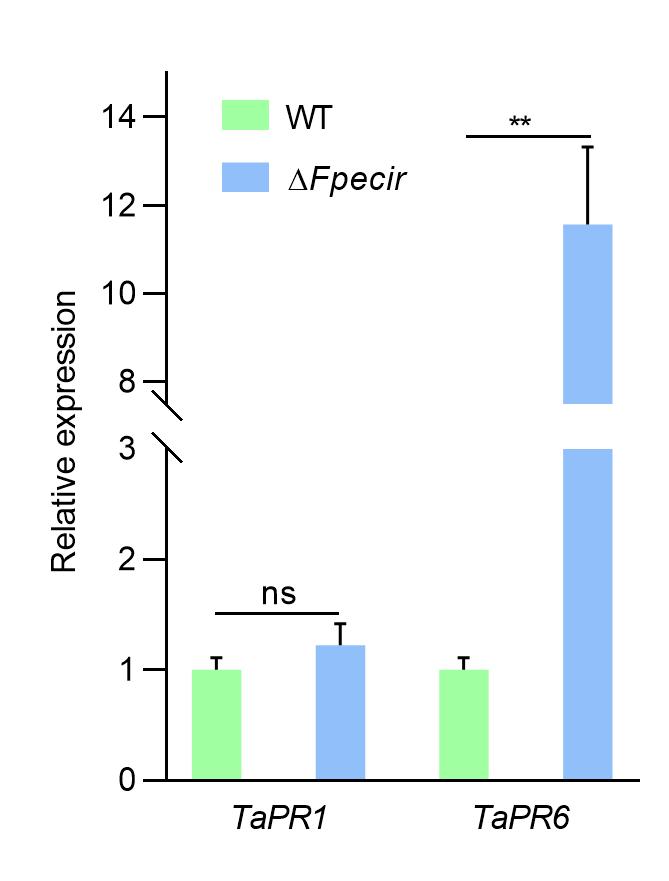


Figure S9. The relative expression of *TaPR1* and *TaPR6*. *TaPR1* belongs to the salicylic acid signaling pathway, and *TaPR6* belongs to the jasmonate acid signaling pathway. Different RNAs were extracted from wheat seedlings inoculated with wild type and Δ*Fpecir* strains. All experiments repeat three times, asterisk indicates significantly differences based on the Student's t-test analysis, *P* < 0.05, experiments were repeated three times.


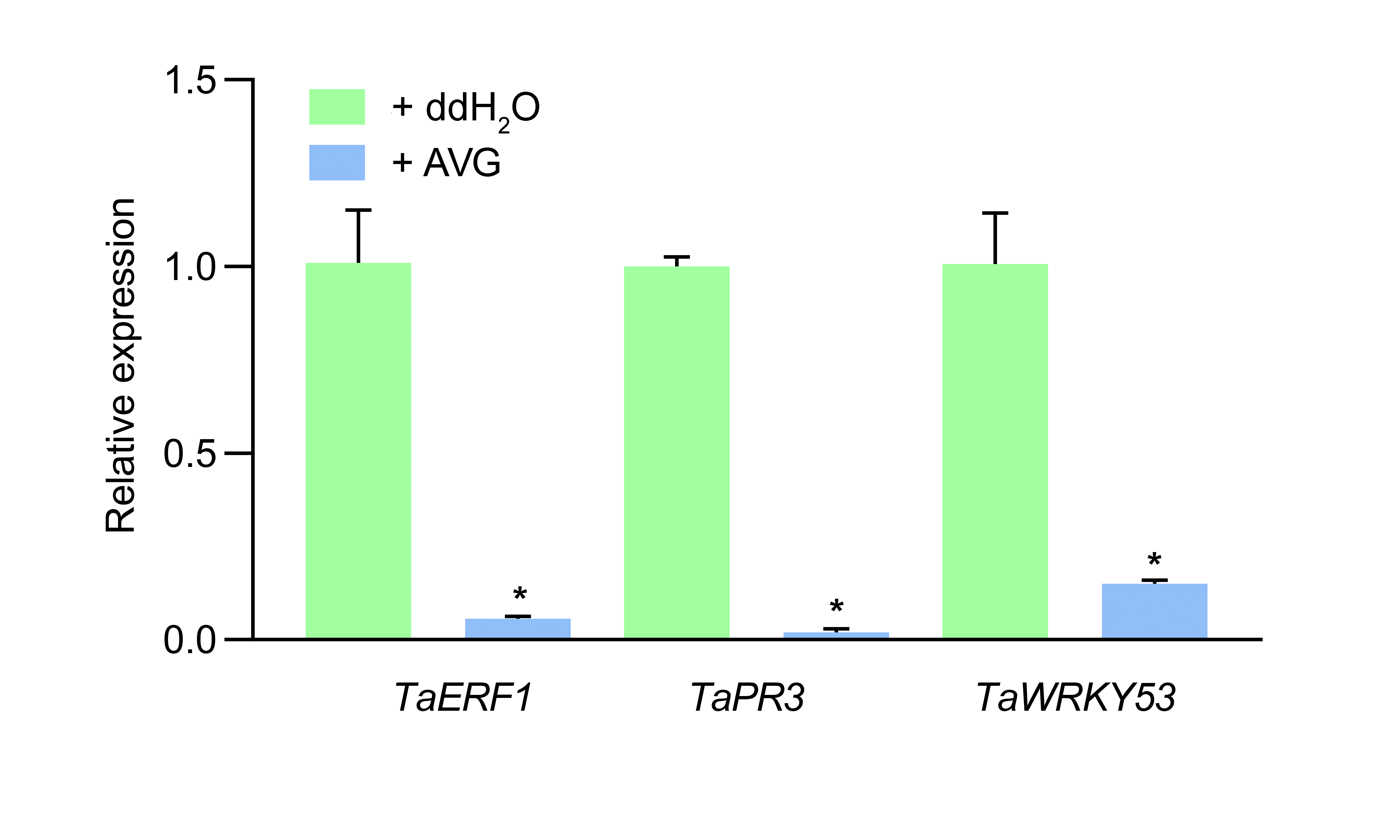


Figure S10. Validation the expression levels of *TaERF1*, *TaPR3*, and *TaWRKY53* using qRT-PCR. Different RNAs were extracted from wheat seedlings which were treated with AVG (+ AVG) or ddH_2_O (- AVG). All experiments repeat three times, asterisk indicates significantly differences based on the Student's t-test analysis, *P* < 0.05, experiments were repeated three times.
